# Identification of a novel type of focal adhesion remodelling via FAK/FRNK replacement, and its contribution to cancer progression

**DOI:** 10.1038/s41419-023-05774-4

**Published:** 2023-04-08

**Authors:** Masatsune Tsujioka, Keisuke Miyazawa, Masaki Ohmuraya, Yoichi Nibe, Tetsuya Shirokawa, Haruko Hayasaka, Tsunekazu Mizushima, Takeshi Fukuma, Shigeomi Shimizu

**Affiliations:** 1 Department of Pathological Cell Biology, Medical Research Institute, Tokyo Medical and Dental University, 1-5-45 Yushima, Bunkyo-ku, Tokyo, 113-8510 Japan; 2 Division of Electrical Engineering and Computer Science, Kanazawa University, Kakuma-machi, Kanazawa, 920-1192 Japan; 3 Nano Life Science Institute (WPI-NanoLSI), Kanazawa University, Kakuma-machi, Kanazawa, 920-1192 Japan; 4 Department of Genetics, Hyogo College of Medicine, Nishinomiya, Hyogo 663-8501 Japan; 5 Department of Life Science, Faculty of Science & Engineering, Kindai University, Higashi-osaka, Osaka, 577-8502 Japan; 6 Department of Gastroenterological Surgery, Graduate School of Medicine, Osaka University Graduate School of Medicine, 2-2 Yamadaoka, Suita, Osaka, 565-0871 Japan

**Keywords:** Focal adhesion, Cancer

## Abstract

Numerous studies have investigated the various cellular responses against genotoxic stress, including those mediated by focal adhesions. We here identified a novel type of focal adhesion remodelling that occurs under genotoxic stress conditions, which involves the replacement of active focal adhesion kinase (FAK) with FAK-related non-kinase (FRNK). FRNK stabilized focal adhesions, leading to strong cell-matrix adhesion, and FRNK-depleted cells were easily detached from extracellular matrix upon genotoxic stress. This remodelling occurred in a wide variety of cells. In vivo, the stomachs of *Frnk*-knockout mice were severely damaged by genotoxic stress, highlighting the protective role of FRNK against genotoxic stress. FRNK was also found to play a vital role in cancer progression, because FRNK depletion significantly inhibited cancer dissemination and progression in a mouse cancer model. Furthermore, in human cancers, FRNK was predominantly expressed in metastatic tissues and not in primary tissues. We hence conclude that this novel type of focal adhesion remodelling reinforces cell adhesion and acts against genotoxic stress, which results in the protection of normal tissues, but in turn facilitates cancer progression.

## Introduction

Cells of living organisms are continuously exposed to various genotoxic stressors. Their DNA is damaged by endogenous stress factors, such as DNA replication errors and reactive oxygen species produced by metabolic processes, as well as by exogenous stress factors, such as ultraviolet light, ionizing radiation, and DNA-damaging agents [1]. Exposure to such factors can result in severe tissue damage and the development of serious diseases, such as cancer. Owing to their biological importance, cellular genotoxic stress responses involving diverse biological phenomena have been analysed, including DNA repair, cell cycle, and cell death, and the important underlying mechanisms appear to be almost fully elucidated [2, 3].

In addition to the well-defined mechanisms of genotoxic stress responses, however, accumulating lines of evidence have also been demonstrated regarding their effects on cell-matrix adhesion, which is mediated by focal adhesions [4–6]. Focal adhesions are composed of integrins, which are heterodimeric, transmembrane adhesion molecules, and various other proteins, such as scaffold, cytoskeletal, and signalling molecules, which directly or indirectly bind to the cytoplasmic tail of integrins [7, 8]. More than 100 focal adhesion component proteins, including focal adhesion kinase (FAK), paxillin, talin, and vinculin, have been identified to date. Many studies have shown that cells subjected to genotoxic stress lose their focal adhesions and detach from the matrix [9, 10]. In contrast, however, several studies have demonstrated the enhancement of cell adhesion by moderate genotoxic stress, as follows. Bacterial genotoxins cause integrin activation in epithelial and mesenchymal cells, resulting in their spread-out morphologies [11]. Endogenous genotoxic stress caused by a mutation within a DNA ligase increases the adhesion and migration abilities of human fibroblasts, and the upregulation of vinculin might be involved in these phenotypes [12]. Of particular interest is that cancer cells that survive radiation therapy have been suggested to promote cancer progression, and a considerable number of studies have demonstrated that cancer cells that have survived irradiation show enhanced invasion activities and increased adhesion abilities. Most of these cells show the increased expression and/or activation of focal adhesion components, such as increased integrin activation and enhanced phosphorylation of FAK [13–16].

The strength of cell-matrix adhesions is based on the number and nature of the focal adhesions, which are largely affected by their compositions and the molecular states of each component. Regarding the integrins, 24 subtypes mediate cell-matrix adhesion differently by their unique selectivity and affinities with their ligands. Talins, which are essential molecules for integrin activation, have two isoforms that show unique properties, including their tissue specificity [7]. FAK, which is a non-receptor tyrosine kinase, is also one of the most important components for the control of cell-matrix adhesion [17, 18]. Adhesion activity is regulated by FAK assembly/disassembly, phosphorylation/dephosphorylation, and proteolysis. The focal adhesion targeting (FAT) domain of FAK directs its localization, and its subsequent autophosphorylation leads to the exposure of the kinase domain. An identical FAT domain exists in another protein, namely, FAK-related non-kinase (FRNK), implicating the regulation of FAK by this protein. However, FRNK is minimally expressed in most body tissues, except for the vascular media, and FRNK-deficient mice show only marginal abnormalities [19, 20]. Therefore, FRNK has not been analysed in detail to date.

The requirement of cell-matrix adhesion for the repair of damaged tissues as well as disease progression, such as cancer metastasis, led us to consider the potential pathophysiological importance of the genotoxic stress-induced enhancement of cell adhesion, which is the opposite to what usually occurs in damaged cells. Nevertheless, the detailed mechanisms involved in the activation of focal adhesion formation upon genotoxic stress are still largely unknown, although a number of studies have shown the increased activation of integrins and the upregulation of focal adhesion components. Therefore, in the present study, we investigated whether and how focal adhesions are modified upon the enhancement of cell adhesion against genotoxic stress, and roles of the modification of focal adhesions in pathophysiological contexts. From our analysis, we identified the biological importance of FRNK. Genotoxic stress induced the replacement of FAK with FRNK in the focal adhesions of various cell lines. This FRNK-involved remodelling reinforced cell-matrix adhesion by stabilizing focal adhesions against genotoxic stress. Physiologically, the modification of focal adhesions protects gastric epithelial tissues from genotoxic stress. In turn, cancer cells, which are subjected to mild genotoxic stress during their development, exploit this system for their progression.

## Materials and methods

### Antibodies and reagents

Antibodies and reagents used in this study are listed in Supplementary Tables S1 and S2.

### Generation of *Frnk*-KO mice using the CRISPR/Cas9 system

*Frnk*-KO mice were generated using the CRISPR/Cas9 system by referring to a previous study that generated *Frnk-*KO mice by targeted disruption of the *Frnk* promoter [19]. *hCas9* mRNA and sgRNAs were generated using pT7-hCas9 and pT7-sgRNA plasmids (kindly provided by Dr. M. Ikawa, Osaka University), respectively [21]. After digestion of pT7-hCas9 with EcoRI, *hCas9* mRNA was synthesized using an in vitro RNA transcription kit (mMESSAGE mMACHINE T7 Ultra Kit, Ambion), according to the manufacturer’s instructions. Two pairs of forward and reverse oligos targeting the 5′-untranslated regions of the *Frnk* gene were annealed and inserted into the BbsI site of the pT7-sgRNA vector. Each target sequence was as follows: Frnk-G1 (5′-CTAGCACCACTTCTCCTTAC-3′) and Frnk-G3 (5′-CCACCAGGCCGCGGCAATGT-3′). After digestion of pT7-sgRNA with XbaI, sgRNAs were synthesized using the MEGAshortscript Kit (Ambion). The precipitated RNAs were dissolved in Opti-MEM I (Life Technologies) at 0.4 µg/ µL.

In vitro fertilization was performed according to the protocol of the Center for Animal Resources and Development at Kumamoto University, Japan (http://card.medic.kumamoto-u.ac.jp/card/english/sigen/manual/onlinemanual.html) using C57BL/6N female mice (Clea-Japan Inc.). Briefly, the prepared RNAs were introduced into fertilized eggs. Fifty prepared eggs were placed in the electrode gap filled with 5 µL of Opti-MEM I containing sgRNA and *hCas9* mRNA, and were subjected to electroporation (25 V, five times). The eggs were then cultured in KSOM medium at 37 °C and 5% CO_2_ in an incubator, and transferred the next day to the oviducts of pseudopregnant females on the day of vaginal plug detection.

*Frnk*-KO mice were housed in a 12-h light /12-h dark cycle at approximately 23 °C and 40% relative humidity at the Laboratory for Recombinant Animals of Tokyo Medical and Dental University, Tokyo, Japan. The Tokyo Medical and Dental University Ethics Committee for Animal Experiments approved all experiments in this study, and all experiments were performed according to their regulations.

### Cell culture

*Bax*/*Bak*-DKO MEFs and *Frnk*-KO MEFs were harvested from *Bax*/*Bak*-DKO and *Frnk*-KO mouse embryos, respectively, at embryonic day 14.5, and immortalized with the SV40 T antigen. *Fak*-KO MEFs were kindly provided by Professor S. Aizawa of RIKEN [22]. *p53*-KO-T cells were isolated from a spontaneous tumour generated in a *p53*-KO mouse. The *Frnk*-KO clones of *p53*-KO-T cells, and murine CT26 colon carcinoma cells [23], were generated using the CRISPR/Cas9 system, as described in Supplementary Fig. S3D. All cell lines used in the present study were cultured in Dulbecco’s modified Eagle’s medium (DMEM) supplemented with 10% foetal bovine serum (FBS), 2 mM L-glutamine, 1 mM sodium pyruvate, 0.1 mM nonessential amino acids, 10 mM HEPES/Na^+^ (pH 7.4), 0.05 mM 2-mercaptoethanol, 100 U/mL penicillin, and 100 µg/mL streptomycin at 37 °C in a humidified atmosphere containing 5% CO_2_ for human cell lines and 10% CO_2_ for the other cell lines.

### Generation of expression constructs and transfection

The transient expression of FAK, the FAK mutants of the cathepsin L cleavage site (FRA, FAR, FAA), FAK deletion mutants with a disrupted enterokinase recognition site, and FRNK were driven by plasmids harbouring each cDNA, which were based on the pEGFP-C2 vector (Clontech) that generates GFP-fusion protein at the N-terminus. Retroviral expression of FAK and FRNK, which are fused with EGFP at the C-terminus and N-terminus, respectively, and EGFP itself was driven by the plasmids based on the modified pMSCV vectors.

For transient transfection of MEFs, plasmid DNA (3 µg) was introduced into cells (1.5 × 10^6^) by electroporation using the Neon^TM^ Transfection System Kit (Invitrogen) according to the manufacturer’s instructions. Transient transfection of human colon cancer cells was performed using Lipofectamine 2000 (Invitrogen) according to the manufacturer’s instruction. For stable transfection, 2 µg of the retroviral vectors was introduced into Plat-E retroviral packaging cells using Lipofectamine 2000 according to the supplier’s protocol. Virus-containing supernatant was collected at 48, 60, and 72 h post-transfection, and cells were infected consecutively three times every 24 h with 4 μg/mL polybrene. Twelve h after the last infection, the medium was replaced with fresh medium. After 24 h, puromycin (10 μg/mL) was added to the medium for selection.

For the experiments of gene silencing, *Bax*/*Bak*-DKO MEFs were transfected with control siRNA (*Silencer* Select Negative Control#1, Invitrogen) and *Nrf2* siRNA (SMARTpool 18024, Dharmacon), respectively, using Lipofectamine RNAiMax (Invitrogen) according to the manufacturer’s instruction. 48 h post-transfection, the transfected cells were used for the experiments of etoposide stimulation.

### Immunoblot analysis

Cells and tissues were lysed in SDS buffer (50 mM Tris-Cl [pH 6.8] and 2% SDS) and RIPA buffer (Nacalai), respectively, followed by sonication. The lysates were then subjected to SDS-PAGE and were transferred to PVDF membranes, which were then incubated with a primary antibody. The immune complexes were detected using an HRP-conjugated secondary antibody and Chemi-Lumi One Super reagent (Nacalai). Original immunoblots are presented in Supplementary Figs. S11–S23.

### Immunofluorescence staining of cultured cells

Cells were fixed in 4% paraformaldehyde and permeabilised with 0.1% Triton-X-100 in PBS. After blocking with 2% FBS in PBS (FBS-PBS) at 4 °C for 30 min, the samples were incubated with a primary antibody in FBS-PBS for 1 h at room temperature or overnight at 4 °C, followed by washing with FBS-PBS three times. The samples were subsequently stained with a fluorescently labelled secondary antibody for 1 h at room temperature and then washed five times with PBS. They were then mounted with ProLong Gold Antifade Reagent with DAPI (Invitrogen). Fluorescence images were observed using a conventional fluorescence microscope (Olympus; IX71) or a confocal microscope (Zeiss; LSM710), and images were obtained using ZEN imaging software.

### RT-PCR

To analyse the expression of the *Frnk* gene in response to genotoxic stress, *Bax*/*Bak*-DKO MEFs [24] were incubated with or without 20 μM etoposide for 14 h. Total RNA was extracted from the cells using an RNA extraction kit (QIAGEN) according to the manufacturer’s instructions. cDNA was synthesized from the total RNA using reverse transcriptase (TOYOBO) and a polyT primer (20-mer), and was used as a template for PCR. The primer pairs used were as follows: the *Fak* mRNA specific region (5′-GATGGCAGCTGCTTATCTTGACC-3′ and 5′-CAGTGCACCTCCTCCGATCGC-3′), the *Frnk* mRNA-specific region (5′-TGTTTCCTGAGTAATTCTGGGTGG-3′ and 5′-TTCTTCTTGCTGCACCTTCTCCTC-3′), and the region common to *Fak* and *Frnk* mRNA (5′-CCTCCAGAAGAGTACGTCCC-3′ and 5′-TCAGTGTGGCCGTGTCTGCC-3′). The PCR products were electrophoresed using a 2% agarose gel.

The RT-PCR method described above was also used to detect the expression of the *FRNK* gene in human colon cancer tissues. Cancer tissues were obtained from Osaka University and from the Bioresource Research Center of Tokyo Medical and Dental University, with approval from their ethics committees, which also approved the protocol to obtain fully informed consent from all participants. Total RNAs were extracted from each tissue sample immersed in RNAprotect Tissue Reagent (QIAGEN). RT-PCR was performed using cDNAs synthesized from the total RNAs and primer pairs, to amplify a specific region of the *FRNK* mRNA (5′-CTCTTTCCTGAGTAATTTTTGGGTGG-3′ and 5′-TCTTCTTGCTGAGCCTTCTCTTCC-3′).

### Cell adhesion and cell spreading assays after the induction of genotoxic stress

To quantify adherent CT26 cells and their derivatives after etoposide treatment, a total of 4 × 10^4^ of each cell type were individually seeded onto 12-well culture plates (Corning) and incubated for 24 h. At 0 h and 24 h following the addition of 100 μM etoposide, cells were washed twice with PBS, and those that remained attached to the surface of the well were collected using a trypsin-EDTA solution (Nacalai) and counted.

To evaluate the cell adhesion ability of *Fak*-KO MEFs expressing GFP, FAK-GFP or GFP-FRNK, subconfluent cells in 6-cm plastic dishes were washed twice with PBS, then treated with 300 μL trypsin-EDTA solution at 37 °C for 5 min. The solution was then removed and the dishes were washed twice with 500 μL of cultured medium. The detached cells in the removed media were counted. The cells remaining attached to the dishes were also collected by pipetting with cultured medium and counted. The adhesion abilities of WT MEFs and *Frnk*-KO MEFs both of which express GFP or GFP-FRNK were evaluated with the same procedure, except that the detachment was induced by the trypsin-EDTA solution diluted twice with PBS for 3 min and the remaining cells were collected using trypsin-EDTA solution with the original concentration. To evaluate the effects of FRNK on cell adhesion in human colon cancer cell lines (DLD1 and HCT116), 1 × 10^4^ cells subjected to transfection with GFP and GFP-FRNK plasmids, respectively, were seeded onto 12-well culture plates (Corning) and incubated for 24 h. Those cells containing GFP-positive and GFP-negative populations were then treated with trypsin-EDTA solution with the same procedure as that of *Fak*-KO MEFs, except that the amount of the trypsin-EDTA solution was 100 μL and the incubation time was 3 min. The GFP signals were analysed for the cells remaining attached. To determine the initial ratios of GFP-positive cells, the seeded cells untreated with trypsin-EDTA solution were examined.

To quantify the spread-out MEFs after etoposide treatment, a total of 1 × 10^4^ MEFs of the *Frnk*-KO and WT littermate mice were seeded onto 24-well culture plates (Corning) and incubated for 24 h. At 0 h and 24 h following the addition of 20 μM etoposide with QVD-OPh, cells were washed twice with PBS, and the number of dark cells, which were judged to be spread-out cells, was counted by observation under phase-contrast microscopy. To binarise the phase-contrast images, the average intensity of a background region absent of cells (20 × 20 pixels) in 8-bit grayscale images were measured using Image J software, and the threshold value for binarisation was set at 95% of the average intensities for the experiment using WT and *Frnk*-KO MEFs treated with etoposide only, and at 90% with etoposide and QVD-OPh. These binarised images were used to measure the spread-out cell areas.

To analyse cell adhesion by the staining of focal adhesions with the anti-paxillin antibody, cells showing more than five focal adhesions, which were defined as discrete signals of more than five pixels long, were quantified.

### Analysis of focal adhesions

The areas and numbers of focal adhesions were measured and counted by extracting focal adhesions from the fluorescence signals of FAK-GFP, GFP-FRNK, or immunostaining of an anti-paxilling antibody, using Hybrid Cell Count software. Cell areas were extracted and measured from the cytosolic fluorescence signals of GFP or immunostaining of the anti-paxilling antibody using Hybrid Cell Count software (KEYENCE).

To evaluate the stability of the focal adhesions via analysing cell retraction, 15-μm square regions containing cell peripheries with the focal adhesion signals were recorded every 3 min. When the peripheries moved out of the square regions by retraction within 30 min, it was defined as ‘retraction events’.

### Quantitative measurements of cell adhesion strength using AFM

Cell detachment experiments were performed referring to the protocol reported previously [25–27], using JPK Nanowizard III ULTRA Speed 2 (Bruker Nano GmbH) equipped with a cantilever with the nominal spring constant of 85 N/m (AC55, Olympus), that was combined with inverted optical microscopy (IX, Olympus). Cells in L15 medium (gibco 21083-027, Thermo Fisher Scientific) were adsorbed on the glass bottom dish (81218-200, ibidi GmbH), and subjected to the analysis at 37 °C. The cantilever deflection signal (*V*_total_) was measured in the lateral tip scanning, in which the scanning speed was 40 µm/s. The measured *V*_total_ was converted to the lateral detachment force (*F*_lat_) using the following equation [26].

$$F_{{{{\mathrm{lat}}}}} = kSV_{{{{\mathrm{total}}}}}\sin ({{\Phi }} + \theta ) \times \cos \left\{ {{{\Phi }} + \theta - 2\tan ^{ - 1}\left[ {\frac{{L - \sqrt {(V_{{\rm{total}}}S)^2 + (L\cos {{\Phi }})^2} }}{{V_{{\rm{total}}}S + L\sin {{\Phi }}}}} \right]} \right\}$$

in which *k*, *S*, and *L* are the spring constant, the sensitivity of the cantilever deflection sensor, and the cantilever length, respectively, *Φ* and *θ* are the angles indicated in Fig. 4F, and *V*_total_ is the cantilever deflection signal.

### Cancer dissemination and tumour formation assay

CT26 cell derivatives (3 × 10^5^ cells) in 150 μL HBSS (Nacalai) were injected into the peritoneal cavity of syngeneic BALB/cCrSlc male mice aged 13 to 18 weeks (Sankyo Labo Service Co., Ltd.). After 10 days, the mice were sacrificed and their mesenteries with disseminated cancer nodules and ascites were collected. To quantify the mesenteric dissemination of each cell line, the weights of mesenteries containing the disseminated cancer cell masses were measured after drying for 24 h.

A total of 3 × 10^5^ *p53*-KO-T cells and 3 × 10^5^ or 1.5 × 10^6^ FRNK-deficient *p53*-KO-T cells were subcutaneously injected into the flanks of syngeneic B6 female mice. After 16 days, the *p53*-KO-T cell tumours were excised, and their weights were measured. For FRNK-deficient cells, tumour formation was analysed after 30 days.

The in vitro sphere formation assay was performed using 3D Tumorsphere Medium XF (PromoCell) according to the manufacturer’s instructions. A total of 5 × 10^4^ cells in the medium were seeded onto 24-well cell-repellent surface plates (Greiner). To count the number of cells composing the spheres, the collected spheres were dispersed by treating with a trypsin-EDTA solution (Nacalai) for 2 h.

### Mouse irradiation

Anaesthetized mice were exposed to 5 Gy of X-rays using an X-ray machine (HITACHI; MBR-1520R-4) at 150 kVp and 20 mA with a 0.3 mm Cu + 0.5 mm Al filter. After 24 h of irradiation, the mice were sacrificed, and various organs and tissues were isolated. The gastric mucosa was collected by scraping the gastric wall with a coverglass (Matsunami; 24 mm × 60 mm).

### Analysis of gastric sections

Mouse stomachs were fixed in 4% paraformaldehyde for 3 h at 4 °C. After washing the samples once with PBS, they were immersed in a 30% sucrose solution in PBS at 4 °C overnight. The specimens were then embedded in OCT compound (Tissue-Tek) in a cryomold (Tissue-Tek) and frozen at −80 °C. The frozen blocks were sliced into 5 to 10-μm sections using a cryostat microtome, mounted on Superfrost Plus microscope slides, and stored in a −80 °C freezer until use.

For HE staining, the sections were first incubated in a haematoxylin solution for 15 min, followed by staining with an eosin solution for 1 min. The sections were then rehydrated by dipping them through a series of graded alcohols (50%, 70%, 80%, 90%, and twice in 100% ethanol), followed by clearing in xylene.

Immunofluorescence staining was performed with the same procedure as that for cultured cells, except that the sections were boiled in 10 mM citric acid beforehand.

TUNEL staining was performed using In Situ Cell Death Detection Kit (Roch) according to the supplier’s protocol.

### Cell invasion assay

The cell invasion assay was applied to two cell lines of human colon cancer (DLD1 and HCT116) transfected with GFP and GFP-FRNK vector, respectively, using Matrigel Invasion Chamber (Corning). A cell culture insert (8 μm pore) containing 2.5 × 10^4^ cells in DMEM lacking any supplements was transferred into a well of the 24-well plate containing cultured medium, which was supplemented with 10% FBS served as a chemoattractant. After incubation of the chamber for 24 h, non-invaded cells on the upper surface of the insert membrane were removed with a cotton swab, and the GFP signal was analysed for transmigrated cells on the lower surface, which were stained with DAPI, using Hybrid Cell Count software (KEYENCE).

### ChIP assay

*Bax*/*Bak*-DKO MEFs treated with or without 20 µM etoposide were fixed in 1% formaldehyde, and the reaction was terminated with 125 mM glycine. The fixed cells were subsequently lysed with cell lysis buffer (5 mM PIPES-KOH [pH 8.0], 85 mM KCl, 0.5% NP-40, and protease inhibitor cocktail [Nacalai]), followed by centrifugation at 2,300 *g* for 5 min. Then, nuclei pellets were lysed with nuclear lysis buffer (50 mM Tris [pH 8.0], 10 mM EDTA, 1% SDS, and protease inhibitor cocktail [Nacalai]), and sonicated using a Branson Sonifier 250. The chromatin solution diluted 5-fold in ChIP dilution buffer (0.01% SDS, 1.1% Triton-X-100, 1.2 mM EDTA, 16.7 mM Tris [pH 8.0], 167 mM NaCl, and protease inhibitor cocktail [Nacalai]) was incubated overnight at 4 °C with specific antibodies. Subsequently, Dynabeads Protein G (Invitrogen) suspended in TE buffer containing 1 µg/μL BSA, 0.2 µg/μL sheared salmon sperm DNA (BioDynamics Laboratory Inc.), and 5% NaN_3_ was added to the solution, which was incubated for another 1 h at 4 °C. The beads were then washed sequentially in wash buffer (0.1% SDS, 1% Triton-X-100, 2 mM EDTA, and 20 mM Tris pH 8.0) containing 150 mM NaCl, wash buffer containing 500 mM NaCl, and LiCl wash buffer (0.25 M LiCl, 1% NP-40, 1% deoxycholate, 1 mM EDTA, and 10 mM Tris [pH 8.0]), followed by extraction twice with elution buffer (1% SDS and 0.1 M NaHCO_3_). After the addition of 0.3 M NaCl and 10 µg of RNase A, the eluates were heated for 4 h at 65 °C to reverse the formaldehyde fixation. The DNA fragments precipitated by ethanol were resuspended and incubated in resuspension buffer (2.5 mM EDTA, 2.5 mM Tris [pH 6.8], 0.2 µg/μL Proteinase K) for 2 h at 45 °C. They were then purified using FastGene Plasmid Mini Kit (Nippon Genetics) and used to amplify the target region by PCR with a primer set (5′-CCTCCATGACCCTACACGTCC-3′ and 5′-CCAAGAAGCCAGGAATATAAATAAGCCC-3′).

### Statistical analysis

For the bar graphs, results were expressed as the mean ± standard deviation (SD; *n* = 3). Comparisons of two datasets were performed using unpaired two-tailed Student *t*-tests using Excel software. In the box plots, the boxes represent the median ± interquartile range, and the whiskers are confidence intervals that denote the 10th to 90th percentiles. Statistical significance was tested using the two-sided Wilcoxon matched-pairs signed-rank test. A *p*-value of less than 0.05 was considered to indicate a statistically significant difference between two groups in both types of statistical analyses.

## Results

### Unique focal adhesions are formed in response to genotoxic stress

**Fig. 1:**
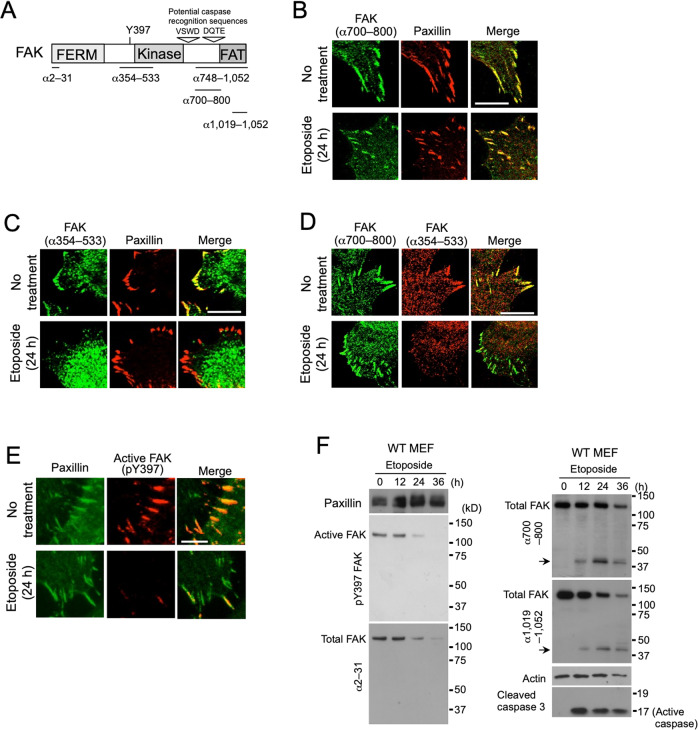
Unusual focal adhesions are formed in cells as a result of genotoxic stress. **A** Schematic representation of FAK showing the positions of the FERM domain, kinase domain, and FAT domain. Regions of the epitopes used for the generation of antibodies that were used in this study are also shown. The 397th tyrosine residue, which is autophosphorylated in active FAK, and the potential cleavage sites of caspases are also indicated. **B**–**D** Confocal fluorescence images of WT MEFs double-immunostained before and after 20 µM etoposide treatment (24 h) with an anti-paxillin antibody (red) and an anti-FAK antibody (α700–800) (green) (**B**), an anti-paxillin antibody (red) and an anti-FAK antibody (α354–533) (green) (**C**), and the two anti-FAK antibodies (α354–533 and α700–800) recognizing its middle (red) and C-terminal region (green), respectively (**D**). **E** Conventional fluorescence images of WT MEFs double-stained with antibodies against active FAK (red) and paxillin (green) before and after 20 µM etoposide treatment (24 h). **F** Immunoblot analysis showing a reduction of FAK but not paxillin, and the generation of a FAK-associated 40-kDa band (arrows) during etoposide treatment of WT MEFs. Active caspase 3 (17 kDa) was generated after etoposide treatment. Actin was used as a loading control. Scale bars, 20 µm (**B**–**D**) and 10 µm (**E**).

As a model to analyse the responses of cell adhesions to genotoxic stress, we treated mouse embryonic fibroblasts (MEFs) with etoposide, a DNA-damaging agent, for 24 h, at which time some populations of cells have become round or detached, whereas other cells are still firmly attached (Supplementary Fig. S1A). We therefore analysed the focal adhesions of these attached cells upon etoposide treatment. Double-immunofluorescence analysis of focal adhesions using antibodies against paxillin and FAK (epitope: amino acids 700–800) (Fig. 1A), both of which are representative components of focal adhesions, demonstrated their colocalization in the focal adhesions of etoposide-treated cells as in untreated cells (Fig. 1B; Supplementary Fig. S1B). Inconsistently, however, when we used a different anti-FAK antibody (epitope: amino acids 354–533), FAK signals were substantially reduced in the focal adhesions of etoposide-treated cells, despite the presence of paxillin signals (Fig. 1C; Supplementary Fig. S1C). Consistently, double immunostaining using the above two anti-FAK antibodies recognizing the middle and C-terminal regions of FAK demonstrated that focal adhesions were only visualized with the C-terminal antibody after etoposide treatment (Fig. 1D; Supplementary Fig. S1D). Furthermore, double immunostaining against paxillin and the active form of FAK (phosho-FAK at tyrosine 397), which is known to localize in focal adhesions, demonstrated the presence of focal adhesions devoid of active FAK in etoposide-treated cells, but not in untreated cells (Fig. 1E; Supplementary Fig. S1E). Therefore, these results led us to infer that genotoxic stress induces the formation of unusual focal adhesions containing C-terminal regions of FAK, instead of active FAK. Immunoblot analysis provided insights into these unusual immunofluorescence staining patterns. Whereas the expression levels of paxillin were constant during etoposide treatment, the expression levels of active and total FAK were progressively reduced (Fig. 1F; left panels). Interestingly, antibodies against the C-terminal regions of FAK (amino acids 700–800 and 1019–1052) demonstrated the generation of an extra band of approximately 40 kDa (Fig. 1F; arrows in the right panels), which was not detected by the N-terminal antibody (Fig. 1F; lower left panel). Taking with the results of immunofluorescence and immunoblot analyses together, we hypothesized that the cells formed a novel type of focal adhesion containing the C-terminal 40-kDa region of FAK instead of active FAK.

### FRNK is expressed in response to genotoxic stress

**Fig. 2:**
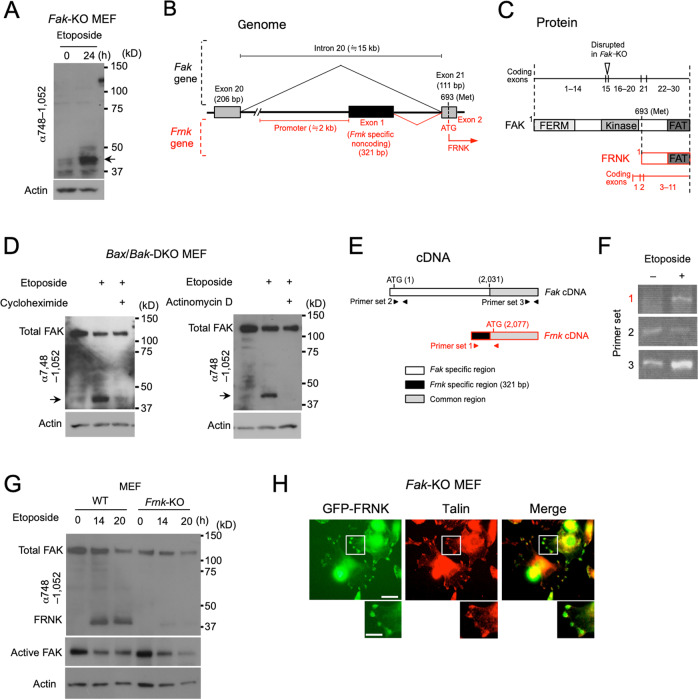
FRNK is expressed in response to genotoxic stress. **A** Immunoblot analysis showing the generation of a FAK-associated 40-kDa band (arrow) even in *Fak*-KO MEFs after etoposide treatment (20 µM, 24 h). **B** Schematic diagram illustrating the genomic region, including exons 20 and 21 of the *Fak* gene (black), and the promoter region and exons 1 and 2 of the *Frnk* gene (red). The initiation codon for the translation of *Frnk* resides in exon 21, and intron 20 contains the promoter and the unique noncoding exon of the *Frnk* gene. Sizes of each exon are indicated in parentheses. **C** Schematic diagram comparing the protein structures of FAK (black) and FRNK (red), as well as demonstrating the positions of their corresponding exons. The 693rd methionine residue of FAK is the start site of FRNK translation, and exon 15 is disrupted in *Fak*-KO MEFs. **D** Immunoblot analysis showing transcription- and translation-dependent generation of the FAK-associated 40-kDa band in *Bax*/*Bak*-DKO MEFs (arrows). For the inhibition of translation and transcription, WT MEFs were treated with 20 µM etoposide for 14 h together with cycloheximide (5 µg/mL) or actinomycin D (0.2 µg/mL). **E** Schematic representation of *Fak* (black) and *Frnk* (red) cDNAs. *Fak*-specific, *Frnk*-specific, and common regions are shown in white, black, and grey, respectively. The ATG initiation codon of *Fak* and *Frnk* are indicated. Numbers in parentheses denote the nucleotide positions from the first nucleotide of *Fak* translation. Arrowheads indicate the locations of the primer sets used to amplify each region. **F** RT-PCR using the primer sets indicated in (**E**), which shows the expression of FRNK mRNA but not FAK mRNA by etoposide treatment in *Bax*/*Bak*-DKO MEFs. **G** Immunoblot analysis showing the absence of the 40-kDa product in *Frnk*-KO MEFs treated with etoposide (20 µM). **H** Fluorescence images showing the localization of GFP-FRNK in focal adhesions. *Fak*-KO MEFs transiently expressing the GFP protein were counterstained with an anti-talin antibody. Magnified images of the boxed regions are shown under each original image. Scale bars, 20 µm (original image) and 10 µm (magnified image). For all immunoblot experiments in Fig. 2, actin was used as a loading control.

We next aimed to characterize the C-terminal 40-kDa region of FAK. We initially predicted that the 40-kDa product resulted from the degradation of FAK, and that FAK is directly cleaved by caspases, which were activated in etoposide-treated MEFs undergoing apoptosis (Fig. 1F; cleaved caspase 3) [9, 28]. However, generation of the 40-kDa product of FAK was not affected by QVD-OPh, a pan-caspase inhibitor, and was not affected in mutant cells simultaneously lacking *Bax* and *Bak* genes (*Bax*/*Bak*-DKO MEFs), which are unable to activate caspases (Supplementary Fig. S2A, B) [24]. We also found potential cleavage sites of cathepsin L and enterokinase in the C-terminal half of FAK (Supplementary Fig. S2C), but all the *Fak*-knockout (KO) MEFs individually expressing FAK mutants, in which these cleavage sites were disrupted, showed the presence of the 40-kDa product upon the treatment of etoposide (Supplementary Fig. S2D, E). More importantly, we detected the 40-kDa product even in *Fak*-KO MEFs treated with etoposide (Fig. 2A; arrow), indicating that it was not a FAK-derived product. These results thus led us to speculate that the 40-kDa product was from the *Frnk* gene, which resides within and shares its coding exons with the *Fak* gene, although it is an independent gene (Fig. 2B). The *Frnk* gene promoter is embedded within intron 20 of the *Fak* gene, which immediately precedes the exon containing the initiation codon of the *Frnk* gene (Fig. 2B). FRNK is translated from the 693rd methionine residue of FAK, and is identical in sequence to the C-terminal region of FAK (Fig. 2C), which is approximately 40 kDa. Note that the *Frnk* gene is intact in the *Fak*-KO MEFs that were analysed, which were generated from a *Fak*-KO mouse lacking only exon 15 of the *Fak* gene (Fig. 2C) [22]. Consistent with our speculation, treatment with either the translation inhibitor cycloheximide or the transcription inhibitor actinomycin D inhibited the expression of the 40-kDa product in etoposide-treated *Bax*/*Bak*-DKO MEFs (Fig. 2D), which enabled more effective sample collection than WT MEFs owing to their higher apoptosis resistance. We subsequently analysed the expression of the *Frnk* gene at the transcriptional level after DNA damage in *Bax*/*Bak*-DKO MEFs, using RT-PCR. To this end, we used a primer set to amplify the 5′-untranslated region specific to *Frnk* mRNA, originating from the unique noncoding exon of the *Frnk* gene (Fig. 2E; primer set 1). *Frnk* mRNA was detected after etoposide treatment (Fig. 2E, F; primer set 1), whereas the expression level of *Fak* mRNA remained unchanged (Fig. 2E, F; primer set 2). When we amplified a region common to *Fak* mRNA and *Frnk* mRNA, the amount of the PCR product was markedly increased by etoposide treatment (Fig. 2E, F; primer set 3), owing to an increase in *Frnk* mRNA. These data confirmed the transcriptional upregulation of the *Frnk* gene upon genotoxic stress. Finally, to determine whether the 40-kDa product represented FRNK, we generated *Frnk*-KO mice by deletion of the 5’-untranslated region of the *Frnk* gene, which includes the noncoding exon, using the CRISPR/Cas9 system (Supplementary Fig. S2F, G). We harvested *Frnk*-KO MEFs from embryos and treated them with etoposide. Deletion of the *Frnk* gene did not affect the expression levels of total or active FAK, but the 40-kDa band was never generated in etoposide-treated *Frnk*-KO MEFs (Fig. 2G), leading us to conclude that FRNK was identical to the 40-kDa product and was expressed by genotoxic stress. FRNK contains the FAT domain, which is a region crucial for the recruitment of proteins to focal adhesions (Fig. 1A), and FRNK was actually recruited to focal adhesions, as shown by the colocalization of GFP-tagged FRNK with talin (Fig. 2H). In addition, after etoposide treatment, expression of the 40-kDa product corresponding to FRNK was accompanied with a reduction in active FAK, as shown in Fig. 1F, and the antibody against both FAK and FRNK (epitope: amino acids 700–800 of FAK) still showed focal adhesion signals, whereas the antibody that detects FAK but not FRNK (epitope: amino acids 354–533 of FAK) scarcely showed focal adhesion signals (Fig. 1D). These results indicated that FAK-FRNK replacement occurred in the focal adhesions in response to genotoxic stress. Note that this type of focal adhesion remodelling is very unique, because it is accomplished by the exchange of two related proteins, and not simply by assembly/disassembly of the components.

### FAK/FRNK replacement supports cell-matrix adhesion as a response to genotoxic stress

**Fig. 3:**
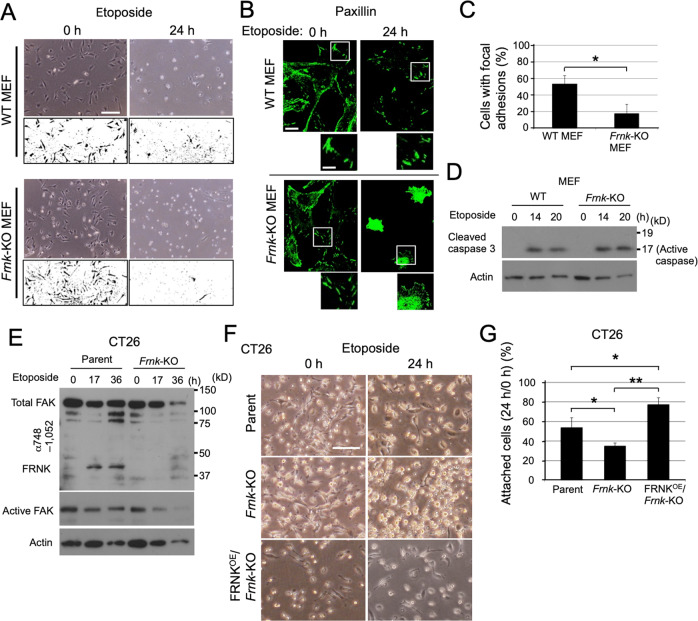
FAK/FRNK replacement supports cell-matrix adhesion against genotoxic stress. **A**–**D** The indicated MEFs were treated with etoposide (20 µM). **A** Representative images showing accelerated cell rounding of *Frnk*-KO MEFs upon etoposide treatment. The phase-contrast images were binarised to extract the spread-out cells, which are visualized as dark cells under the microscope, and are shown under each original image. Scale bar, 200 µm. **B** Confocal images of paxillin staining in WT and *Frnk*-KO MEFs before and after etoposide treatment. Magnified images of the boxed regions are shown under each original image. In etoposide-treated *Frnk*-KO MEFs, focal adhesion signals were faint, but instead, dense fluorescence signals were evenly distributed throughout the cytoplasm of the shrunken cells. Scale bars, 20 µm (original image) and 10 µm (magnified image). **C** Graph showing the fractions of cells demonstrating focal adhesion signals that fulfil the criteria (see Materials and Methods) at 24 h after etoposide treatment. **D** Immunoblot analysis showing equivalent expression levels of active caspase 3 in WT and *Frnk*-KO MEFs after etoposide treatment. **E**–**G** The indicated CT26 derivatives were treated with etoposide (100 µM). **E** Immunoblot analysis showing the absence of FRNK in *Frnk*-KO CT26 cells. **F** Representative phase-contrast images showing the morphological alterations in the indicated CT26 derivatives 24 h after etoposide treatment. Scale bar, 200 µm. **G** Graph showing the fractions of cells remaining attached 24 h after etoposide treatment in the indicated CT26 derivatives. All data in the graphs are shown as means ± standard deviations (SD; *n* = 3). The two-sided Student’s *t* test was used for statistical analysis. *p < 0.05, **p < 0.01 in (**C**, **G**). For all immunoblot experiments in Fig. 3, actin was used as a loading control.

We next analysed the role of FAK/FRNK replacement in focal adhesions upon genotoxic stress. Although *Frnk*-KO MEFs looked healthy under normal culture conditions, they became round faster than MEFs from their WT littermates upon etoposide treatment (Fig. 3A). This difference was clearly demonstrated by binarising the phase-contrast images; cells that were spread out were visualized as dark cells under the microscope (Fig. 3A). *Frnk*-KO MEFs also tended to lack paxillin signals in focal adhesions upon etoposide treatment (Fig. 3B, C), indicating the rapid loss of focal adhesions. The accelerated cell rounding of *Frnk*-KO MEFs was not a result of the difference in apoptosis efficiency; the levels of caspase 3 activation were comparable between etoposide-treated WT and *Frnk*-KO MEFs (Fig. 3D), and QVD-OPh treatment did not alter the phenotype of *Frnk*-KO MEFs (Supplementary Fig. S3A–C). Therefore, our results demonstrate that FAK/FRNK replacement supports cell attachment against genotoxic stress, which occurs by a mechanism independent of apoptosis.

To investigate the generality of these focal adhesion responses, we used CT26 murine colon carcinoma cells, which show an increase in FRNK levels and decrease in active FAK levels in response to genotoxic stress (Fig. 3E, “parent”). FRNK-deficient CT26 cells, which were generated using the CRISPR/Cas9 system (Supplementary Fig. S3D, E), were less attached than their parental CT26 cells after etoposide treatment (Fig. 3F, G), which was similar to our observation in MEFs. The overexpression of FRNK in the *Frnk*-KO strain (FRNK^OE^/*Frnk*-KO CT26) ameliorated the loss of cell attachment against etoposide treatment (Fig. 3F, G; Supplementary Fig. S3F). These data confirmed the genotoxic stress-induced expression of FRNK and its contribution to cell attachment. Importantly, the upregulation of FRNK induced by genotoxic stress can be observed in a wide variety of cell lines, such that 11 out of the 35 cell lines analysed expressed FRNK in response to genotoxic stress (Supplementary Table S3).

### FRNK stabilizes focal adhesions

**Fig. 4:**
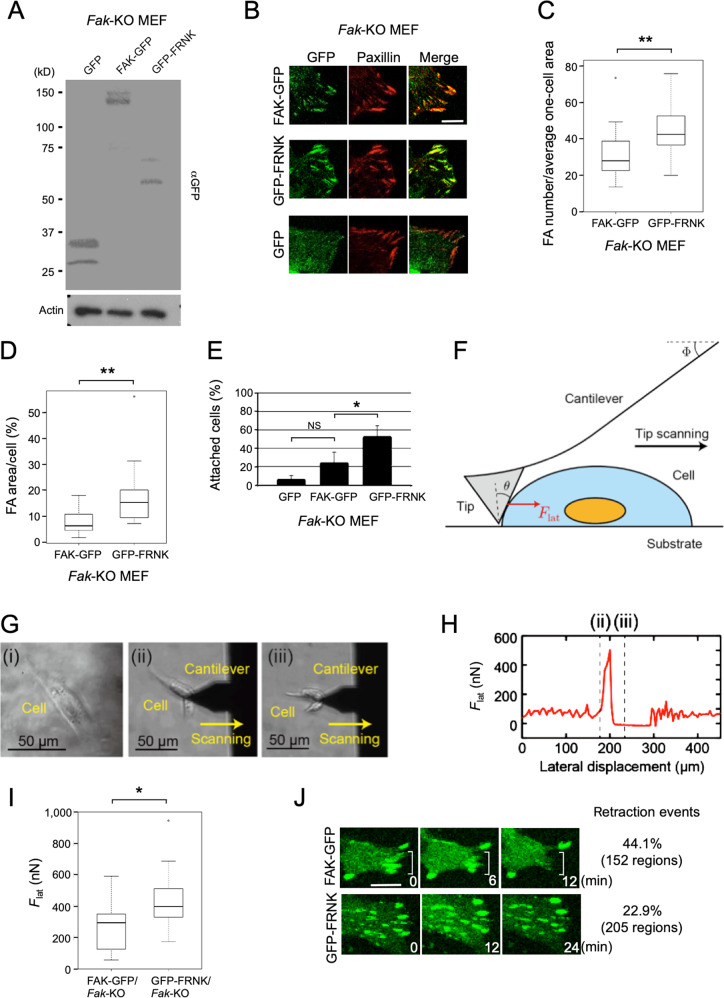
FRNK stabilizes focal adhesions. **A** Immunoblot analysis showing comparable expression levels of GFP, FAK-GFP, and GFP-FRNK, which are detected by an anti-GFP antibody (αGFP**)**, in the indicated stable transformants of *Fak*-KO MEFs. Actin was used as a loading control. **B** Confocal fluorescence images of the respective transformants of *Fak*-KO MEFs counterstained with an anti-paxillin antibody. **C**, **D** Box plots showing the focal adhesion (FA) number per average one-cell area (2,750 µm^2^) (**C**) and the percentage of focal adhesion area per cell (**D**) in the *Fak*-KO MEFs expressing FAK-GFP or GFP-FRNK. **E** Graph showing cell fractions of the *Fak*-KO MEF transformants remaining attached after trypsin-EDTA treatment. All data in the graph are shown as means ± SD (*n* = 3). The two-sided Student *t*-test was used for statistical analysis. *p < 0.05, NS: no significance. **F** Schematic illustration to explain the interactions between the AFM tip and a cell in the lateral detachment force (*F*_lat_) measurement. The angles *Φ* and *θ* were measured for the calculation of *F*_lat_ (see Materials and Methods). **G** Phase-contrast images showing the process of the lateral scanning experiment. A GFP-FRNK-expressing cell in (i) was subjected to the scanning, as shown in (ii) and (iii). **H** *F*_lat_ changes in the experiment of (**G**). Figure 4G (ii) and (iii) were obtained at the time indicated by the dotted lines (ii) and (iii) in (**H**), respectively. **I** Box plots showing *F*_lat_ measured in the *Fak*-KO MEF transformants. **J** Time-lapse fluorescence imaging showing representative behaviours of focal adhesions and cell edges in each *Fak*-KO MEF transformant. Frequencies of retraction events accompanied with the dissolution of focal adhesion signals are shown on the right side. Scale bars, 10 µm (**B**) and 15 µm (**J**). In all box plots, boxes represent the median ± interquartile range, and whiskers are confidence intervals that denote 10th to 90th percentiles. Significance was tested using the two-sided Wilcoxon matched-pairs signed-rank test. *p < 0.05, **p < 0.01.

We next sought to characterize FAK-containing and FRNK-containing focal adhesions under normal culture conditions, using *Fak*-KO MEFs individually expressing either FAK-GFP or GFP-FRNK, as well as their control cells expressing GFP (Fig. 4A). FAK-GFP and GFP-FRNK but not GFP itself localized in the focal adhesions counterstained with an anti-paxillin antibody (Fig. 4B; Supplementary Fig. S4A). The numbers and areas of the focal adhesion signals per cell were larger in the cells expressing GFP-FRNK than in those expressing FAK-GFP, despite their comparable expression levels (Fig. 4A, C, D). We also analysed focal adhesions visualized by an anti-paxillin antibody in the three cell lines. Consistent with the analysis using GFP signals, cells expressing GFP-FRNK showed larger numbers and areas of focal adhesions than cells expressing FAK-GFP and cells expressing GFP, which displayed no significant difference in the number and area of focal adhesions (Supplementary Fig. S4B, C). Even in WT and *Frnk*-KO MEFs, both of which constitutively express endogenous FAK, the forced expression of GFP-FRNK showed essentially the same effects on focal adhesions as in *Fak*-KO MEFs (Supplementary Figs. S5A–D and S6A–D).

We subsequently investigated the effects of the forced expression of FRNK on cell adhesion. When adhesion abilities were compared among the three cell lines derived from *Fak*-KO MEFs by treating them with trypsin-EDTA, GFP-FRNK-expressing cells were most resistant to detachment, with no significant difference between the other two cell lines (Fig. 4E). Similarly, WT and *Frnk*-KO MEFs overexpressing GFP-FRNK were more resistant to the trypsin-EDTA treatment than their control cells expressing GFP (Supplementary Figs. S5E and S6E). We next measured the adhesion strength of *Fak*-KO MEFs expressing FAK-GFP and those expressing GFP-FRNK more directly, using atomic force microscopy (AFM). In these experiments, the AFM tip was scanned laterally to the middle of a cell to detach it (Fig. 4F, G), and the lateral detachment force (*F*_lat_) was measured during this tip lateral scanning (Fig. 4F–H). We found that *F*_lat_ values were significantly larger in cells expressing GFP-FRNK than in those expressing FAK-GFP (Fig. 4I), demonstrating that GFP-FRNK-expressing cells adhered to the substrate more strongly. We finally investigated the association between focal adhesions and cell attachment by analysing the cell peripheries, where focal adhesions were abundant and local detachment/attachment activities were dynamic, using time-lapse fluorescence microscopy. *Fak*-KO MEFs expressing FAK-GFP more frequently showed retraction of the cell edges containing the focal adhesion signals during a 30-min observation than those expressing GFP-FRNK, whereas cells expressing GFP-FRNK tended to show persistent focal adhesions and more static peripheries than those expressing FAK-GFP (Fig. 4J). We therefore conclude that FRNK plays a specific role in stabilizing focal adhesions, as a component of focal adhesions. This newly identified function is the cause of the increase in the area and number of focal adhesions in FRNK-expressing cells, leading to their firm attachment.

### Nrf2 induces the expression of FRNK upon genotoxic stress

**Fig. 5:**
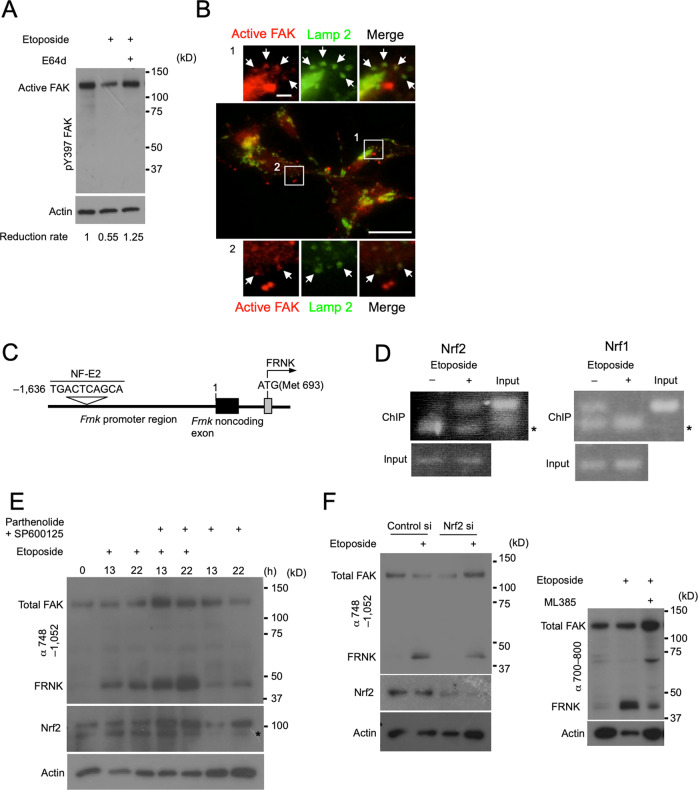
Nrf2 drives the expression of FRNK upon genotoxic stress. **A**, **B** *Bax*/*Bak*-DKO MEFs were treated with etoposide (20 µM). **A** Immunoblot analysis demonstrating the suppression of etoposide-induced active FAK reduction by E64d (80 µg/mL), which is a broad lysosomal protease inhibitor. Band intensities of active FAK were normalized by actin used as a loading control, and the reduction rates are shown below the blots. **B** Immunofluorescence images showing the colocalization of active FAK and lamp2, a lysosome marker, by cotreatment with etoposide and E64d (80 µg/mL). Magnified images of the boxed regions are shown in the top and bottom panels. Arrows indicate the colocalization of the two proteins. Scale bars, 30 µm (original image) and 10 µm (magnified images). **C** Schematic diagram illustrating the area around the promoter region of the *Frnk* gene. The grey and black boxes indicate the exon containing the initiation codon of *Frnk* and the noncoding exon, respectively. The consensus NF-E2 binding sequence (the NF-E2 element), in which the first nucleotide is located 1636 bp upstream of the transcription start site of the *Frnk* gene, is also indicated. **D**–**F** *Bax*/*Bak*-DKO MEFs were treated with etoposide (20 µM). **D** The ChIP assay demonstrating Nrf2 binding to the NF-E2 element in place of Nrf1 in an etoposide-dependent manner. Interaction of the indicated proteins with the NF-E2 element was electrophoretically analysed. Asterisks indicate nonspecific bands. **E** Immunoblot analysis showing that the upregulation of Nrf2 by cotreatment with 5 µM parthenolide and 25 µM SP600125 coincided with the increase in the etoposide-induced expression of FRNK. The asterisk indicates nonspecific bands. **F** Immunoblot analysis showing that the etoposide-induced expression of FRNK was diminished, owing to the reduced expression of Nrf2 by gene silencing (left panels) and by blockade of the nuclear translocation of Nrf2 with 20 µM ML385 (right panels). Actin was used as a loading control for all immunoblot experiments in this figure.

We next investigated the mechanism of the FAK/FRNK replacement, using *Bax*/*Bak*-DKO MEFs. A reduction in active FAK level upon etoposide treatment was not the consequence of FRNK expression, because it was also observed in *Frnk*-KO cells (Figs. 2G, 3E). This reduction was found to be mediated by lysosomal degradation, because it was blocked by E64d, a lysosomal protease inhibitor (Fig. 5A), and because active FAK colocalized with lysosomes upon the simultaneous treatment of etoposide and E64d (Fig. 5B). Regarding the upregulation of FRNK, we searched the promoter region of the *Frnk* gene, and focused on the consensus binding sequence for the NF-E2 transcription factor complex (the NF-E2 element) (Fig. 5C) [29], because NF-E2 is known to respond to genotoxic stress [30–33]. Expectedly, the chromatin immunoprecipitation (ChIP) assay demonstrated that Nrf2, a component of NF-E2, bound to the NF-E2 element in an etoposide-dependent manner (Fig. 5D). Conversely, Nrf1, another Nrf protein, was found to constitutively bind to the NF-E2 element, and etoposide treatment substantially reduced this binding activity (Fig. 5D), which is reasonable because Nrf1 and Nrf2 are mutually exclusive. We next investigated whether Nrf2 is involved in the expression of FRNK, by perturbing Nrf2 expression and function. Treatment of *Bax*/*Bak*-DKO MEFs with parthenolide and SP600125, which are compounds that reportedly enhance Nrf2 expression [33, 34], did not clearly upregulate Nrf2, although they modestly induced the expression of FRNK (Fig. 5E). However, the administration of these compounds together with etoposide somehow markedly increased the expression of Nrf2, and concomitantly upregulated FRNK (Fig. 5E). Conversely, the downregulation of Nrf2 by gene silencing attenuated the induction of FRNK expression upon etoposide stimulation (Fig. 5F; left panels). Furthermore, when cells were simultaneously treated with etoposide and ML385, which blocks the nuclear translocation of Nrf2, the induction of FRNK was even more attenuated (Fig. 5F; right panels). These findings demonstrated that genotoxic stress upregulates FRNK in an Nrf2-dependent manner.

### FAK/FRNK replacement protects gastric tissues from genotoxic stress

**Fig. 6:**
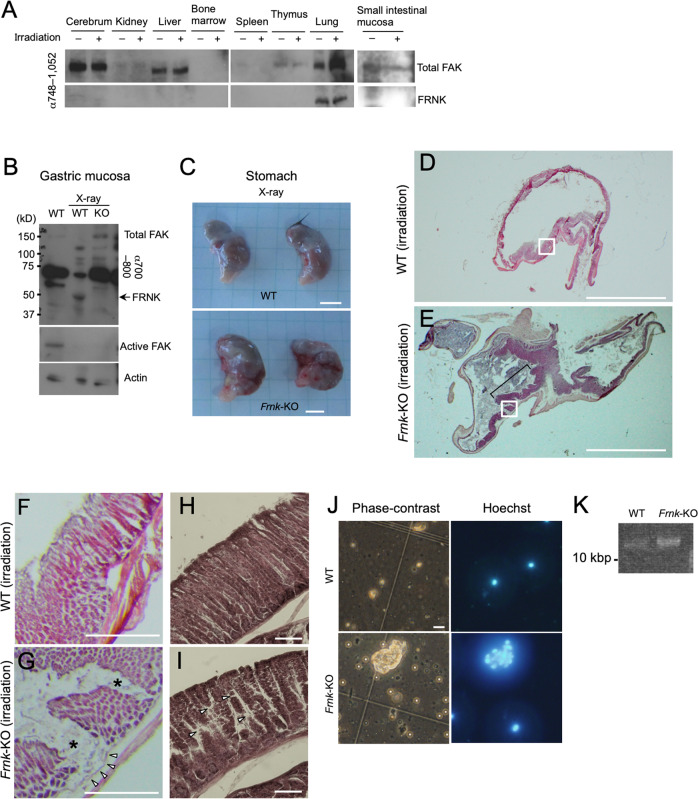
FAK/FRNK replacement protects gastric tissues from genotoxic stress. **A** WT mice were or were not irradiated with 5 Gy, and analysed for the expression of FAK and FRNK in each tissue after 24 h. **B** WT and *Frnk*-KO mice were irradiated with 5 Gy, and analysed for the expression of FAK and FRNK in the gastric mucosa after 24 h. Among the various tissues, the gastric mucosa was found to express FRNK and downregulate active FAK in response to irradiation (**B**). Note that FRNK was constitutively expressed in the lungs as already reported, but the level was not affected by irradiation (**A**). Actin was used as a loading control. **C**–**K** WT and *Frnk*-KO mice were irradiated with 5 Gy and their gastric mucosa were analysed after 24 h. **C** Macroscopic images of the stomachs of WT and *Frnk*-KO mice after irradiation. Scale bar, 5 mm. **D**–**I** HE staining of sliced sections of the irradiated stomachs of WT and *Frnk*-KO mice. **F**, **G** are magnified images of the boxed regions in (**D**, **E**), respectively. In (**E**), the bracket indicates the uneven thickness of the gastric mucosa of *Frnk-*KO mice. In (**G**), asterisks and white arrowheads indicate partial loss of the epithelial layer and the intact muscularis mucosa, respectively, in the stomachs of *Frnk*-KO mice. In (**I**), the white arrowheads indicate gaps between aligned epithelial cells in the gastric mucosa of *Frnk-*KO mice. Scale bars, 5 mm (**D**, **E**), 0.5 mm (**F**, **G**), and 100 µm (**H**, **I**). **J** Phase-contrast images showing dissociated cells from the mucosal layers after irradiation and their corresponding fluorescence images showing nuclear staining with Hoechst 33342. **K** Estimation of the number of dissociated cells from the mucosal layers by visualizing their extracted DNA by EtBr staining after agarose gel electrophoresis. The size of the stained band was more than 10 kbp.

We next analysed the physiological role of FRNK using *Frnk*-KO mice. Although we did not find any apparent defects in *Frnk*-KO mice, the in vitro functions of FRNK in the genotoxic stress response prompted us to analyse the effects of exposure of the mice to X-ray irradiation. Among the multiple organs and tissues analysed for the expression of FRNK 24 h after X-ray irradiation (Fig. 6A), the gastric mucosa showed substantial irradiation-dependent FRNK accumulation, as well as a reduction in active FAK level (Fig. 6B), leading us to focus our analysis on gastric tissue. Stomachs from *Frnk*-KO mice exposed to irradiation tended to be flabby and have irregular shapes, and were red compared with those from WT mice (Fig. 6C). Analysis of sliced sections of the irradiated stomachs stained with haematoxylin and eosin (HE) showed that the stomachs of *Frnk*-KO mice were highly deformed (Fig. 6D, E; Supplementary Fig. S7A, B), and demonstrated characteristics of acute gastritis, unlike their WT counterparts (Fig. 6F–I; Supplementary Fig. S7C–F). The thickness of the mucosal layer was uneven, and a partial loss of the epithelial layer was detected (Fig. 6E; bracket and 6G; asterisks). The muscularis mucosa was intact in the epithelium-depleted region (Fig. 6G; white arrowheads), indicating that the damage was limited to the epithelium. Microscopic observation revealed a sparse appearance of the *Frnk*-KO epithelium (Fig. 6I), which resulted partially from gaps between the aligned epithelial cells (Fig. 6I, arrowheads). The results of the histological analysis suggested that the epithelial cells of *Frnk*-KO mouse stomachs were easily detached from their underlying matrix. When we collected the stomach contents from irradiated WT and *Frnk*-KO mice, we observed more cell masses in the contents from the stomachs of *Frnk*-KO mice than from those of WT mice (Fig. 6J). To estimate the quantity of the dissociated cells, we extracted genomic DNA from the detached cells within the stomach contents, subjected the genomic DNA to agarose gel electrophoresis, and stained the gel with ethidium bromide (EtBr). A stained band was observed only in the samples from the stomachs of *Frnk*-KO mice (Fig. 6K), indicating the presence of a larger amount of dissociated cells in the stomachs of *Frnk*-KO mice than WT mice.

If cells are easily dissociated, increased apoptosis is expected to follow owing to the induction of anoikis, a type of apoptosis triggered by cell detachment. In fact, the *Frnk*-KO specimens more frequently showed apoptotic cells, defined by active caspase 3 immunofluorescence and TUNEL staining, than those of WT mice (Supplementary Fig. S7G; arrows and bracket). Immunoblot analysis confirmed the increase in active caspase 3 in the irradiated stomachs of *Frnk*-KO mice compared with those of WT mice (Supplementary Fig. S7H). Cumulatively, these results demonstrate that FRNK is involved in genotoxic stress tolerance of the gastric mucosa, by reinforcing the attachment between epithelial cells and their underlying matrix.

### FAK/FRNK replacement supports cancer progression

**Fig. 7:**
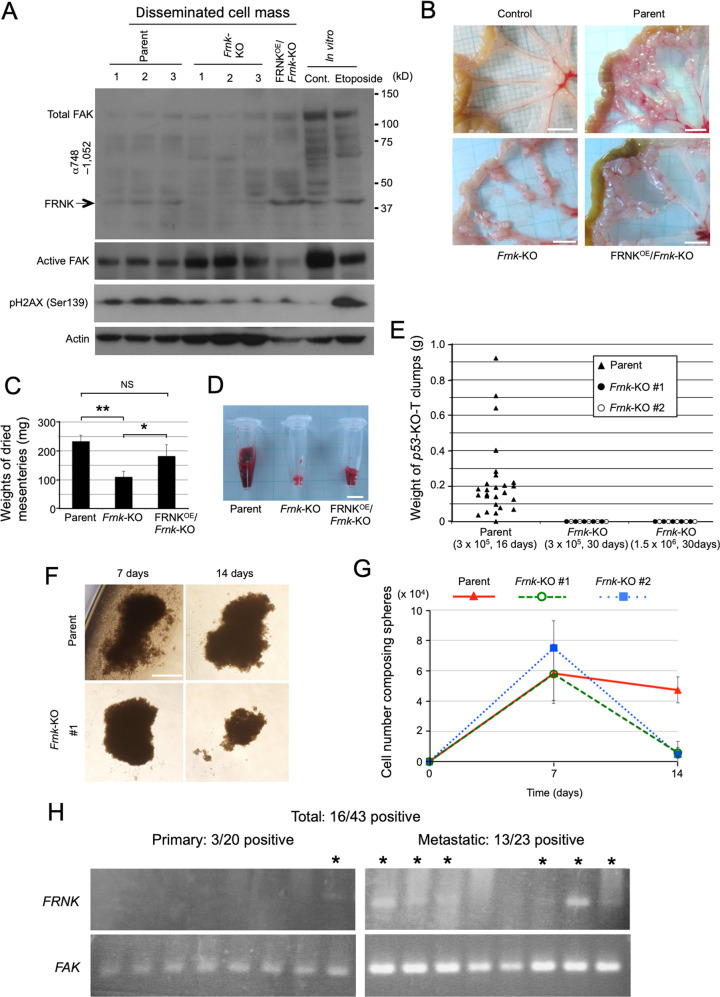
FAK/FRNK replacement accelerates cancer progression. **A**–**D** CT26 cell derivatives (3 × 10^5^ cells) were injected into the peritoneal cavity of male syngeneic BALB/c mice, and analysed after 10 days. **A** Immunoblot analysis showing the expression levels of the indicated proteins in three independent peritoneal disseminated cell masses of parental and *Frnk*-KO CT26 strains, as well as one cell mass of the FRNK^OE^/*Frnk*-KO strain. Cultured CT26 cells that were untreated or stimulated with 100 µM etoposide were used as negative and positive controls, respectively. Actin was used as a loading control. **B** Macroscopic images showing tumour mass dissemination in the mesenteries of mice injected with parental, *Frnk*-KO, and FRNK^OE^/*Frnk*-KO CT26 cells. **C** Graph indicating the dry weights of the mesenteries of mice injected with parental, *Frnk*-KO, and FRNK^OE^/*Frnk*-KO CT26 cells. All data in the graphs are shown as means ± SD (*n* = 3). The two-sided Student’s *t* test was used for statistical analysis. *p < 0.05, **p < 0.01, NS: no significance. **D** Photo showing bloody ascites taken from the peritoneal cavities of mice injected with parental, *Frnk*-KO, and FRNK^OE^/*Frnk*-KO CT26 cells. **E** Plots showing the weights of tumour masses subcutaneously formed by the indicated *p53*-KO-T derivatives. **F**, **G** *P53*-KO-T cell derivatives (5 × 10^4^ cells) were 3D-cultured for the indicated days. **F** Phase-contrast images showing the appearance of spheres formed by *p53*-KO-T cell derivatives in vitro. **G** Graph showing the number of cells forming spheres at 7 days and 14 days after the induction of sphere formation. **H** Gel electrophoresis following RT-PCR showing the expression levels of *FRNK* and *FAK* mRNA in representative primary and metastatic human cancer tissues. The asterisks indicate samples showing the expression of *FRNK* mRNA. Scale bars, 5 mm (**B**, **D**), 100 µm (**F**).

We next analysed the involvement of FRNK in cancer dissemination, as cancer cells show an association between cell adhesion ability and their progression [35–38], and are continuously exposed to moderate genotoxic stress during their progression [39]. We intraperitoneally injected CT26 derivatives into male syngeneic BALB/c mice, and analysed their disseminated tumour cell masses. Immunoblot analysis using lysates prepared from three independent cell masses of parental cells demonstrated a band of gamma-H2AX phosphorylation (pH2AX), which is a marker for double-stranded breaks in DNA, and FRNK expression was also apparent (Fig. 7A, “parent”), consistent with the genotoxic stress response under in vitro conditions. We also confirmed the absence of and overexpression of FRNK in the disseminated cell masses of *Frnk*-KO and FRNK^OE^/*Frnk*-KO cells, respectively (Fig. 7A). The overexpression of FRNK substantially reduced active FAK in FRNK^OE^/*Frnk*-KO cell masses, indicating that FAK/FRNK replacement occurs also in this situation. Nevertheless, substantial amounts of active FAK remained in the parental cell masses, indicating that FAK is only partially or temporarily replaced with FRNK in these tissues. Ten days after their injection, parental CT26 cells were extensively disseminated throughout the peritoneal cavity, whereas *Frnk*-KO cells showed substantially reduced gross-cell masses, and this phenotype was considerably reduced in *Frnk*-KO cells re-expressing FRNK (Fig. 7B). The reduction in cancer dissemination in *Frnk*-KO cells was confirmed by the weight of their mesenteries (Fig. 7C). The amounts of bloody ascites clearly reflected the different degrees of dissemination among the CT26 derivatives (Fig. 7D; Supplementary Fig. S8A). We thus propose that partial and/or temporal FAK/FRNK replacement in focal adhesions facilitates the dissemination of cancer cells, probably via the reinforcement of cell adhesion under conditions of genotoxic stress.

As another approach to investigate the effects of FRNK expression on cancer progression, we used a murine cancer cell line, namely, *p53-*KO-T. *P53* is the most frequently mutated tumour suppressor gene in humans, and *p53-*KO-T was isolated and established from a spontaneous cancer in a *p53*-KO mouse. We generated two clones of *Frnk*-deficient *p53-*KO-T by the same strategy as used to generate *Frnk*-KO CT26 cells (Supplementary Fig. S3D). The *p53-*KO-T strain, but not the two mutant clones, expressed FRNK in response to genotoxic stress in vitro (Supplementary Fig. S8B), demonstrating that p53 is not essential for FRNK expression. The three cell lines were subcutaneously injected into female syngeneic B6 mice. Tumour formation of the parental strain was apparent in 27 of the 28 injected areas 16 days after inoculation (Fig. 7E; Supplementary Fig. S8C). However, despite an equivalent proliferation rate in vitro (Supplementary Fig. S8D) and the same number of injected cells, neither of the two FRNK-depleted strains formed tumour masses, even after 30 days (five injected areas/clone) (Fig. 7E). Even when we injected a five times greater number of cells, tumour masses were not formed (five injected areas/clone) (Fig. 7E). The expression of FRNK as well as pH2AX was detected in the parental tumours using immunoblot analysis (Supplementary Fig. S8E, F), demonstrating that FRNK is essential for the tumour formation of *p53*-KO-T cells. To analyse the impairment in the formation of tumour cell masses in FRNK-deficient *p53-*KO-T cells, we performed the in vitro sphere formation assay. The *Frnk*-KO strains formed spheres that were indistinguishable in appearance and size from those of the parental strain within seven days (Fig. 7F, G). Unlike the parental strain, however, the mutant strains were not able to maintain their sphere size, which became smaller with time (Fig. 7F, G). Expectedly, FRNK and pH2AX were expressed in the parental spheres (Supplementary Fig. S8G). We thus suggest that the cell adhesion ability of the *Frnk*-KO strains is insufficient for the growth and maintenance of their spheres, owing to the loss of FRNK-containing focal adhesions under genotoxic stress conditions, which results in their inability to form dysplastic tumour masses in vivo.

Finally, we investigated whether the *FRNK* gene is expressed in human cancer cells. The expression of *FRNK* mRNA was analysed by RT-PCR of mRNAs extracted from excised colon cancer tissues from human patients. *FRNK* mRNA was detectable in 16 out of the 43 samples (37.2%). Notably, when restricted to metastatic colon cancer tissues, 13 out of the 23 samples (56.5%) were positive for *FRNK* mRNA signals (Fig. 7H; Supplementary Table S4). Considering the functions of FRNK that have been clarified up to this point, the expression of FRNK may accelerate metastasis via the enhancement of cell attachment in some types of human cancer cells.

## Discussion

The present study showed for the first time that focal adhesions are remodelled by FAK/FRNK replacement in response to genotoxic stress, to support cell adhesion against this stress (Supplementary Fig. S9). FRNK was found to be expressed in the gastric mucosa of X-ray irradiated mice, to maintain tissue integrity. These findings led us to conclude that a major function of FRNK is to adapt cells and tissues to genotoxic stress conditions by reinforcing cell adhesion.

Although FAK is known to be regulated in various ways, including assembly/disassembly and phosphorylation/dephosphorylation [17, 18], FAK/FRNK replacement is a novel type of modification of focal adhesions, which is accomplished by the exchange of the two related proteins, and not simply by the assembly or disassembly of the components. FRNK was previously considered to be an endogenous inhibitor of FAK, owing to its unique structure [40]. However, our findings indicate that FRNK substitutes for FAK as a focal adhesion component upon genotoxic stress, to play specific pathopysiological roles in cell adhesion, rather than inhibiting FAK. This mechanism has not been previously identified in experiments using the artificial expression of FRNK. Interestingly, genotoxic stress concomitantly induced FAK degradation and FRNK expression. These two events are mutually independent, because FAK degradation and FRNK expression occurred in FRNK-deficient cells and FAK-deficient cells, respectively. Some upstream factors of the genotoxic stress responses may orchestrate this synchronous mechanism, which presently remains unclear.

We suggest that FAK/FRNK replacement enhances cell adhesion by stabilizing focal adhesions, which is mainly based on the results regarding the characteristics of focal adhesions containing FAK and FRNK, and the strength of cell adhesion measured by AFM. We believe that the stabilization of focal adhesions by FRNK is reasonable, because FRNK would still be able to act as a scaffold protein within the focal adhesions, as it binds other focal adhesion components, such as paxillin and p130Cas [41], and it lacks the kinase activity of FAK, which is required for the disassembly of focal adhesions [42, 43]. This firm attachment mediated by FAK/FRNK replacement is crucial, because the transient reinforcement of cell adhesions can avoid the uncontrolled loss of cell adhesions in damaged cells and resultant tissue disruption.

In vivo, FRNK is predominantly expressed in smooth muscle cells (SMCs) composing the vascular media, with its expression barely detectable in other tissues [20, 29]. *Frnk*-KO mice showed delayed differentiation from synthetic to contractile SMCs during the growth and remodelling of blood vessels, which, nevertheless, did not result in apparent defects in the developed vascular systems. As the vascular media is continuously exposed to mechanical strain caused by blood pressure, strong and stable cell-matrix adhesion mediated by FRNK may contribute to the formation and maintenance of the tissues. Similar to the X-ray irradiation of gastric tissue that was performed in the present study, experiments involving severe perturbation of the vascular system may clarify additional important functions of FRNK in the tissues.

The enhanced adhesion of cells mediated by FRNK in reaction to genotoxic stress would be advantageous to cancer cells, as they are continuously exposed to moderate genotoxic stress [39], and many reports have shown an association between cell attachment ability and tumour progression [35–38]. We therefore hypothesized that FRNK is involved in tumour progression. Cancer cells tested in the mouse model in this study demonstrated increased dissemination into the abdominal cavity (CT26), and tumorigenesis (*p53*-KO-T) owing to FRNK expression. Similarly, the prometastatic roles of FRNK were suggested in humans from the results of FRNK expression analysis in cancer tissues. FRNK is thought to support cell attachment in reaction to genotoxic stress under these in vivo conditions. Given the complete lack of growth of FRNK-deficient *p53*-KO-T cells in vivo, FRNK-mediated cell adhesion may be crucial for the dysplastic growth of cancer cells in the early phase. Consistently, spheres of FRNK-deficient *p53*-KO-T cells formed in vitro were disassembled before they grew to submillimeter diameters.

As our study using human colon cancer tissues showed that FRNK was more frequently expressed in metastatic than primary tissues, FRNK expression may promote cancer metastasis through increased adhesion. When we tested an in vitro assay for cell invasive migration, that mimics a part of metastasis (see Materials and Methods), using two human colon cancer cell lines (DLD1 and HCT116) overexpressing FRNK, the expression of GFP-FRNK, which was confirmed to facilitate cell adhesion in both cell lines, activated invasive migration in the DLD1 cell line (Supplementary Fig. S10A, B). In contrast, however, HCT116 cells showed the decreased activity of invasive migration (Supplementary Fig. S10B). Strong cell adhesion provides a large traction force for cell migration, whereas its adverse effect on cell detachment resists cell locomotion [44, 45]. Therefore, the effects of enhanced adhesion on cell migration would be affected by some cell characteristics, which should be taken into consideration to clarify the relationship between FRNK expression and metastasis.

## Supplementary information

Supplemental Material

aj-checklist

## Data Availability

All data generated and analysed during the current study are available from the corresponding authors upon reasonable request.
