# Supplementary material for: Identification of a novel type of focal adhesion remodelling via FAK/FRNK replacement, and its contribution to cancer progression
Source: Cell Death Dis. 2023 Apr 8;14(4):256. doi: 10.1038/s41419-023-05774-4 (PMC10082854; doi:10.1038/s41419-023-05774-4)
Supplement: Supplementary file 1 — Supplemental Material [file 41419_2023_5774_MOESM1_ESM.docx]

**Supplementary Figures**

**
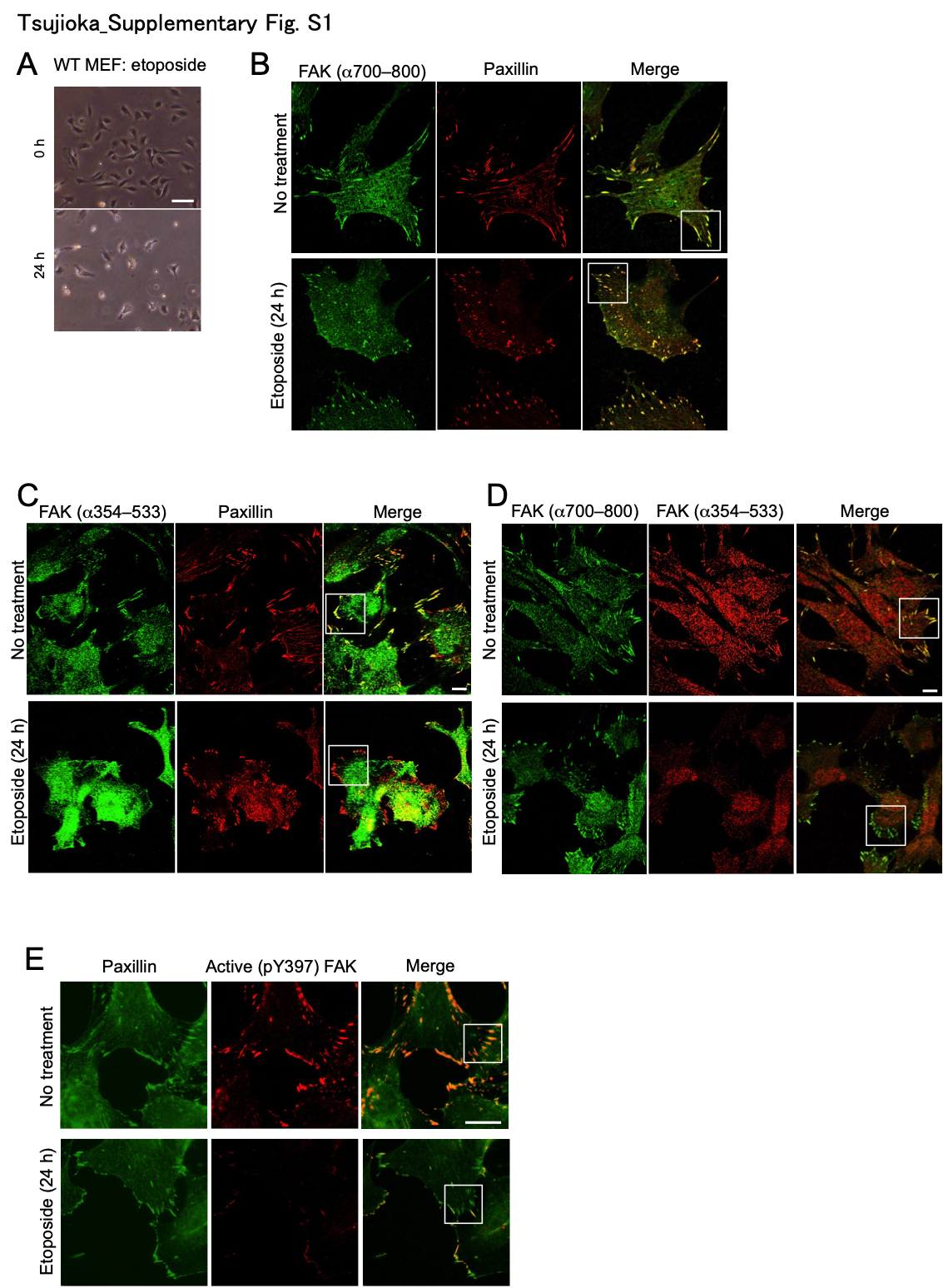
**

**Supplementary Fig. S1. Formation of unusual focal adhesions upon genotoxic stress.**

WT MEFs were treated with etoposide (20 µM) for 24 h. **A** Phase-contrast images showing morphological changes of WT MEFs upon genotoxic stress. Scale bar, 100 µm. **B–E** Full images of the fluorescence images shown in Fig. 1B–E, which were magnified images of the boxed regions. Scale bars, 20 µm

**
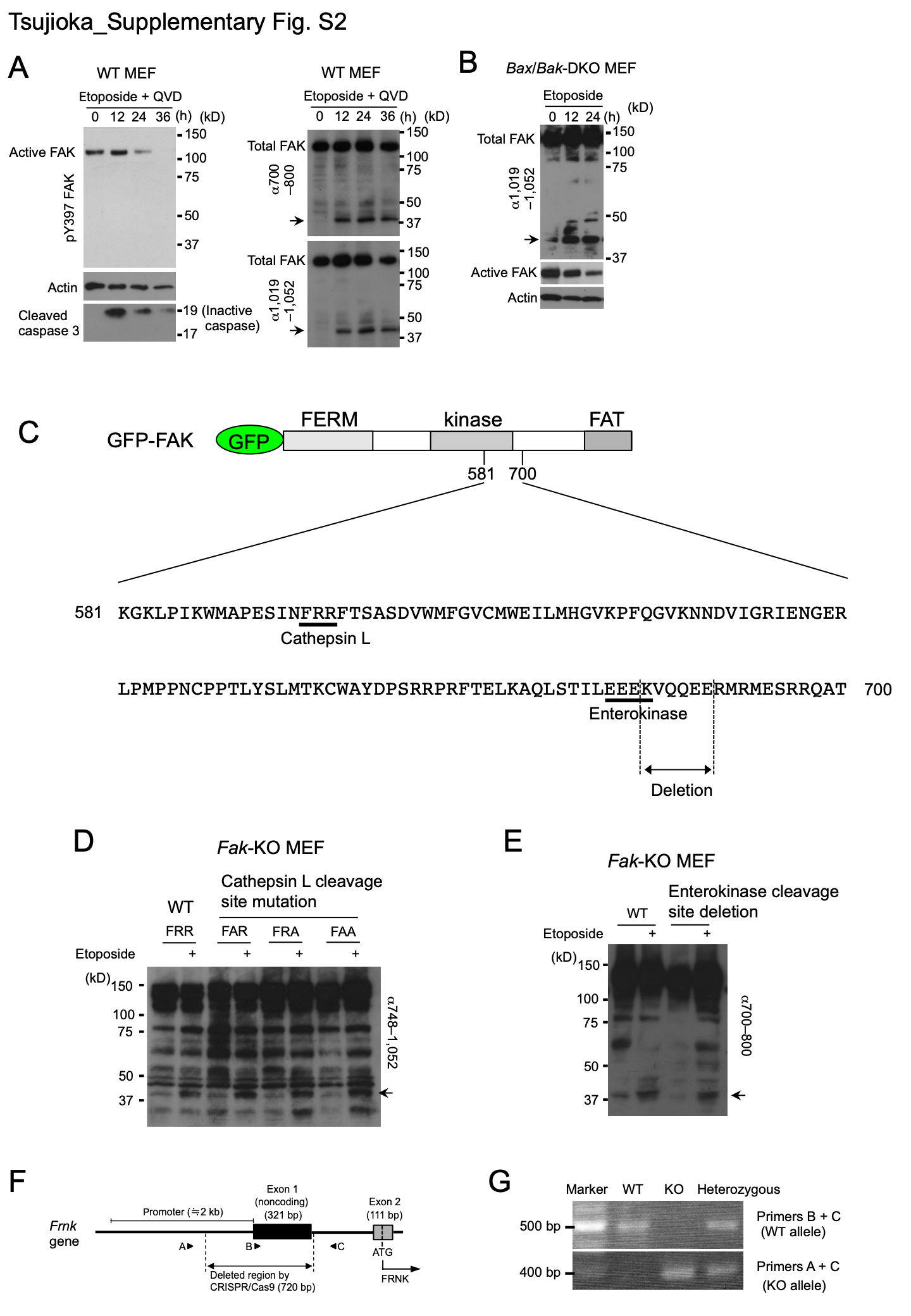
**

**Supplementary Fig. S2. Expression of FRNK in response to genotoxic stress.**

**A, B** The indicated MEFs were treated with etoposide (20 µM). (**A**) Immunoblot analysis showing that the inhibition of caspases by QVD-OPh (100 µM) does not affect the generation of the 40-kDa band (arrows) after etoposide treatment. Caspase 3 was cleaved but only the inactive (19 kDa) form was generated. (**B**) Immunoblot analysis showing generation of the FAK-associated 40-kDa band (arrow) in *Bax*/*Bak-*DKO MEFs. Actin was used as a loading control. **C** Schematic diagram showing potential cleavage sequences of cathepsin L and enterokinase (underlined). ‘Deletion’ indicates the deleted region of the FAK mutant used in (**E**). **D** The cathepsin L target site is not cleaved in etoposide-treated cells. *Fak*-KO MEFs were transiently transfected with 3 µg of plasmids encoding FAK, or its three individual mutants in which the cathepsin L target site was disrupted (‘FAR’, ‘FRA’, and ‘FAA’). Cells were then exposed to 20 µM etoposide for 26 h, and the generation of the 40-kDa product was analysed by immunoblotting using the indicated FAK antibody. The arrow indicates the 40-kDa product. **E** The enterokinase target site was not cleaved in etoposide-treated cells. A similar experiment to (**D**) was performed by introducing a different *Fak* mutant gene, in which the enterokinase target site was disrupted. *Fak*-KO MEFs expressing this *Fak* mutant still yielded the 40-kDa product, as indicated by the arrow. **F** Schematic diagram illustrating the genomic region including the promoter region, and exons 1 and 2 of the *Frnk* gene. The sizes of each exon are indicated in parentheses. The diagram also shows the deleted region of the *Frnk* gene in *Frnk*-KO mice. Black arrowheads indicate the locations of the primers used for genotyping. **G** Genotyping by genomic PCR showing the generation of heterologous and homologous mice for KO of the *Frnk* gene. As illustrated in (**F**), a 495-bp DNA fragment is amplified from the WT allele by genomic PCR using primers B and C, whereas a 396-bp fragment is amplified from the mutant allele using primers A and C.

**
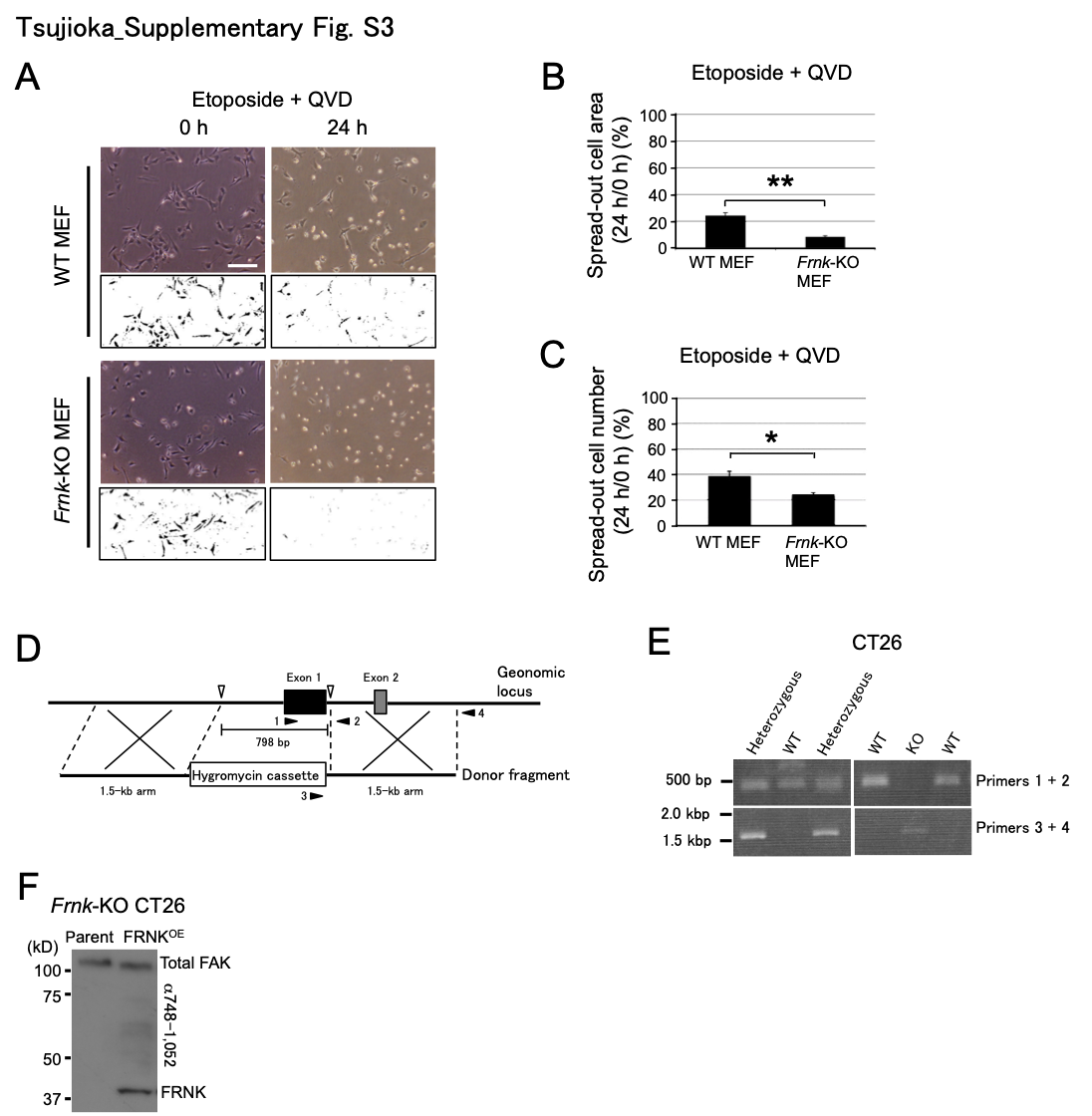
**

**Supplementary Fig. S3. Significance and generality of FAK/FRNK replacement in cells upon genotoxic stress.**

**A–C** The indicated MEFs were treated with etoposide (20 µM) in the presence of QVD-OPh (100 µM). (**A**) Representative images showing that QVD-OPh does not inhibit the accelerated cell rounding of *Frnk*-KO MEFs upon etoposide treatment. Phase-contrast images were binarised as in Fig. 3A. Scale bar, 200 µm. (**B, C**) Graphs showing the ratios of the spread-out cell areas (**B**) and numbers (**C**) at 24 h with respect to those at 0 h. All data in the graphs are shown as means ± SD (n = 3). The two-sided Student *t*-test was used for statistical analysis. *: *p* < 0.05, **: *p* < 0.01. **D** Schematic diagram showing the *Frnk* gene KO strategy using the CRISPR/Cas9 system in cultured cells. The genomic locus around the target region and the donor fragment for homologous recombination are shown. The black and grey boxes indicate exons 1 and 2 of the *Frnk* gene, respectively. White arrowheads indicate the sites where double stranded breaks are expected to occur. As illustrated, the donor fragment is comprised of the hygromycin resistance gene cassette and genomic fragment arms, and the hygromycin cassette is expected to replace a 798-bp genomic region. Black arrowheads indicate the locations of the primers used for genotyping. **E** Genotyping by genomic PCR in CT26 cells. As illustrated in (**D**), genomic PCR using primers 1 and 2 amplified a 495-bp fragment almost covering exon 1, whereas primers 3 and 4 were used to amplify an approximately 1.5-kbp fragment that is produced only when the desired replacement occurs. WT and KO derivatives only showed the band amplified by primers 1 and 2 and primers 3 and 4, respectively. The heterozygous derivative showed both bands. **F** Immunoblot analysis showing the re-expression of FRNK in FRNK^OE^/*Frnk*-KO CT26 cells.

**
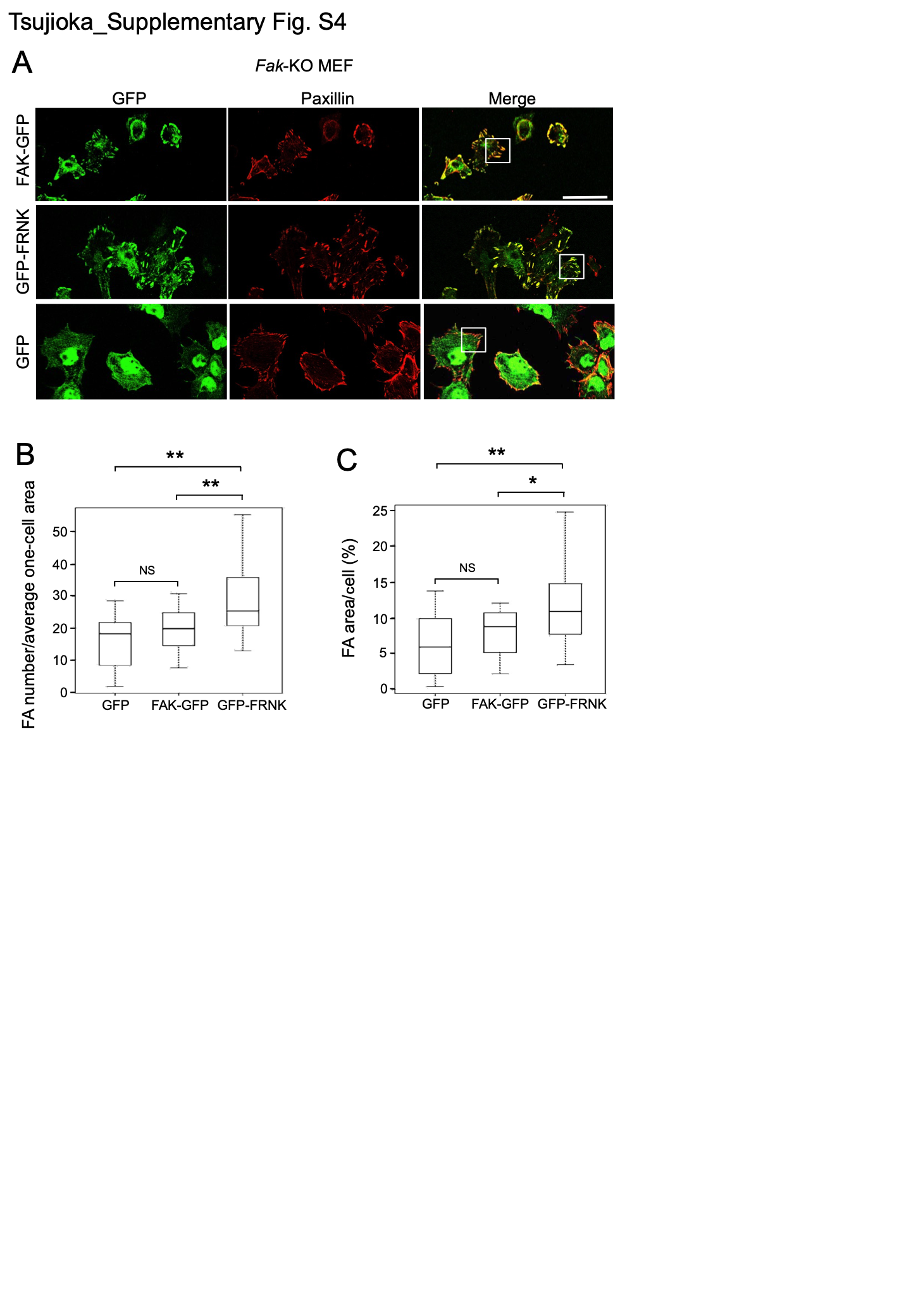
**

**Supplementary Fig. S4. Analysis of focal adhesions in *Fak*-KO MEF transformants.**

Full images of the fluorescence images shown in Fig. 4B, which were magnified images of the boxed regions. Scale bar, 50 µm. **B, C** Box plots showing the focal adhesion numbers per average one-cell area (2,750 µm^2^) (**B**), and the percentage of focal adhesion area per cell (**C**) in the *Fak*-KO MEFs expressing either GFP, FAK-GFP, and GFP-FRNK, which were subjected to paxillin antibody staining for focal adhesion analysis. Boxes represent the median ± interquartile range, and whiskers are confidence intervals that denote 10th to 90th percentiles. Significance was tested using the two-sided Wilcoxon matched-pairs signed rank test. *: *p* < 0.05, **: *p* < 0.01, NS: no significance


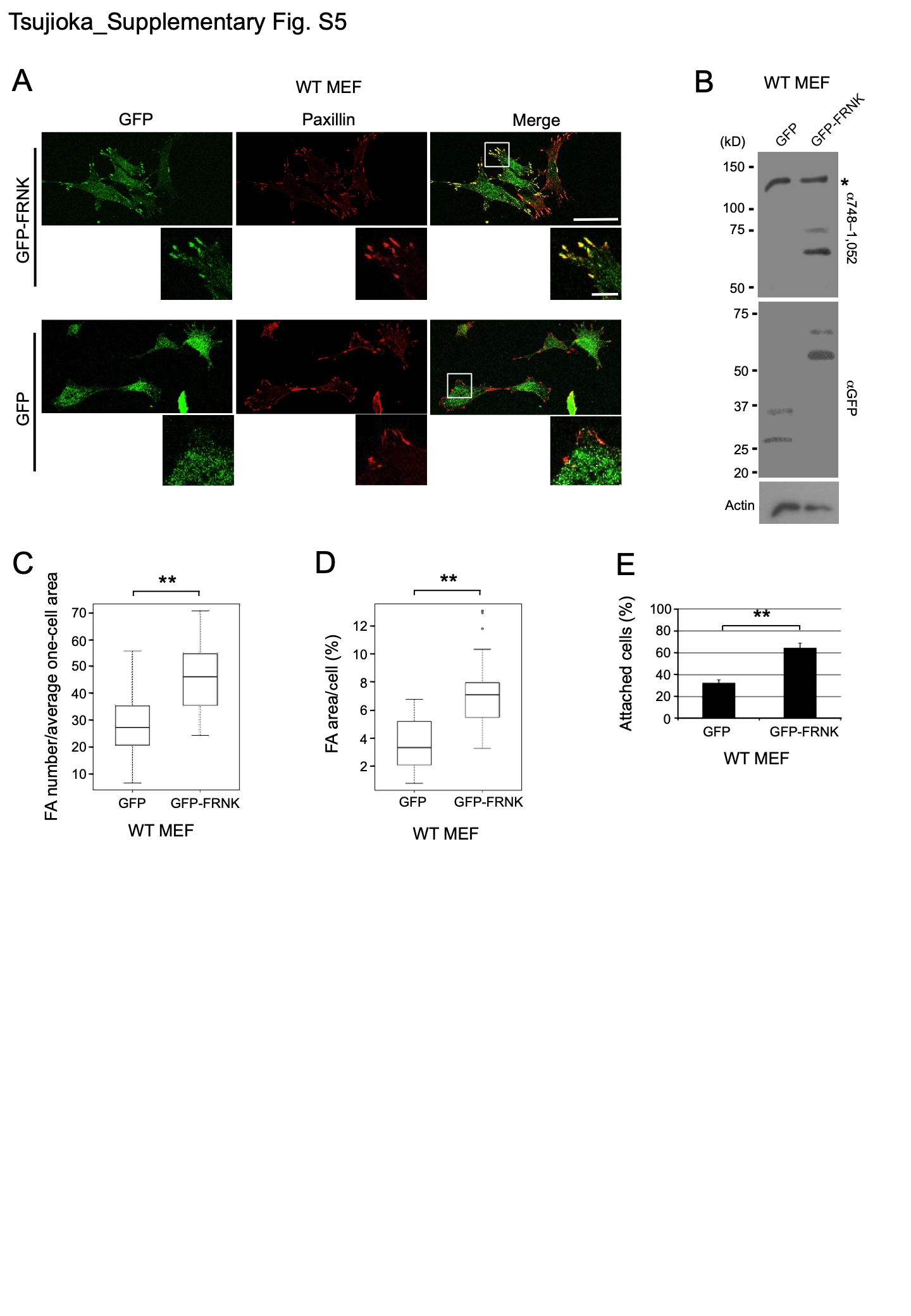


**Supplementary Fig. S5. Effects of the expression of GFP-FRNK on cell adhesion in WT MEFs.**

**A** Confocal fluorescence images of WT MEFs stably expressing GFP and GFP-FRNK, respectively, counterstained with an anti-paxillin antibody. Magnified images of the boxed regions are shown under each original image. Scale bars, 50 µm (original image) and 10 µm (magnified image). **B** Immunoblot analysis using the indicated antibodies, which shows the expression of GFP and GFP-FRNK in the indicated transformants of WT MEFs. The asterisk represents the expression of endogenous FAK. Actin was used as a loading control. **C, D** Box plots showing the focal adhesion numbers per average one-cell area (2,750 µm^2^) (**C**), and the percentage of focal adhesion area per cell (**D**) in the WT MEF transformants, which were subjected to staining with the paxillin antibody, for focal adhesion analysis. Boxes represent the median ± interquartile range, and whiskers are confidence intervals that denote 10th to 90th percentiles. Significance was tested using the two-sided Wilcoxon matched-pairs signed rank test. **: *p* < 0.01. **E** Graph showing cell fractions of the WT MEF transformants remaining attached in the trypsin-EDTA exposure. All data in the graph are shown as means ± SD (n = 3). The two-sided Student *t*-test was used for statistical analysis. **: *p* < 0.01


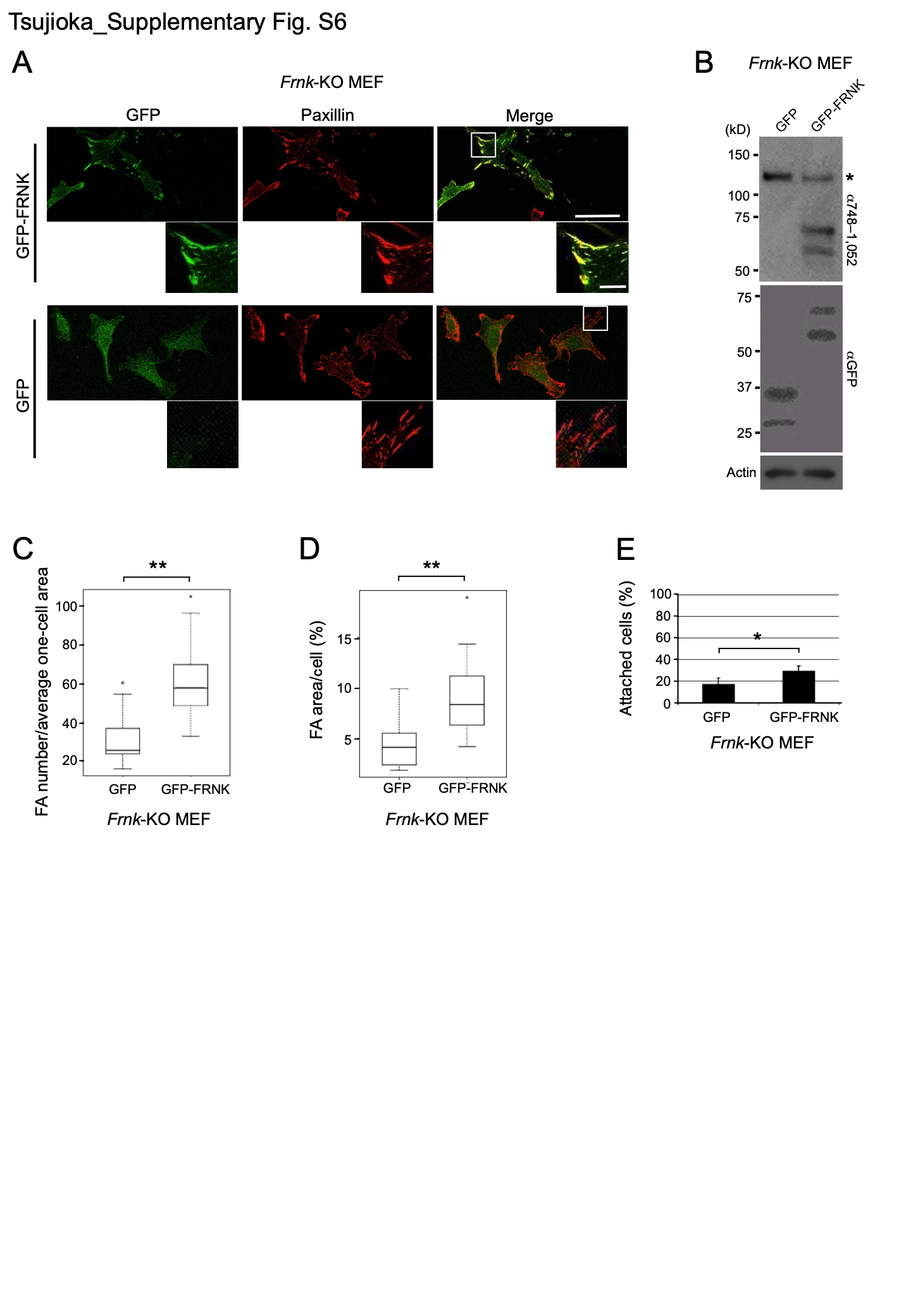


**Supplementary Fig. S6. Effects of the expression of GFP-FRNK on cell adhesion in *Frnk*-KO MEFs.**

**A** Confocal fluorescence images of *Frnk*-KO MEFs stably expressing GFP and GFP-FRNK, respectively, counterstained with an anti-paxillin antibody. Magnified images of the boxed regions are shown under each original image. Scale bars, 50 µm (original image) and 10 µm (magnified image). **B** Immunoblot analysis using the indicated antibodies, which shows the expression of GFP and GFP-FRNK in the indicated transformants of *Frnk*-KO MEFs. The asterisk represents the expression of endogenous FAK. Actin was used as a loading control. **C, D** Box plots showing the focal adhesion numbers per average one-cell area (2,750 µm^2^) (**C**) and the percentage of focal adhesion area per cell (**D**) in the *Frnk*-KO MEF transformants, which were subjected to staining with the paxillin antibody for focal adhesion analysis. Boxes represent the median ± interquartile range, and whiskers are confidence intervals that denote 10th to 90th percentiles. Significance was tested using the two-sided Wilcoxon matched-pairs signed rank test. **: *p* < 0.01. **E** Graph showing cell fractions of the *Frnk*-KO MEF transformants remaining attached after the trypsin-EDTA treatment. All data in the graph are shown as means ± SD (n = 3). The two-sided Student *t*-test was used for statistical analysis. *: *p* < 0.05


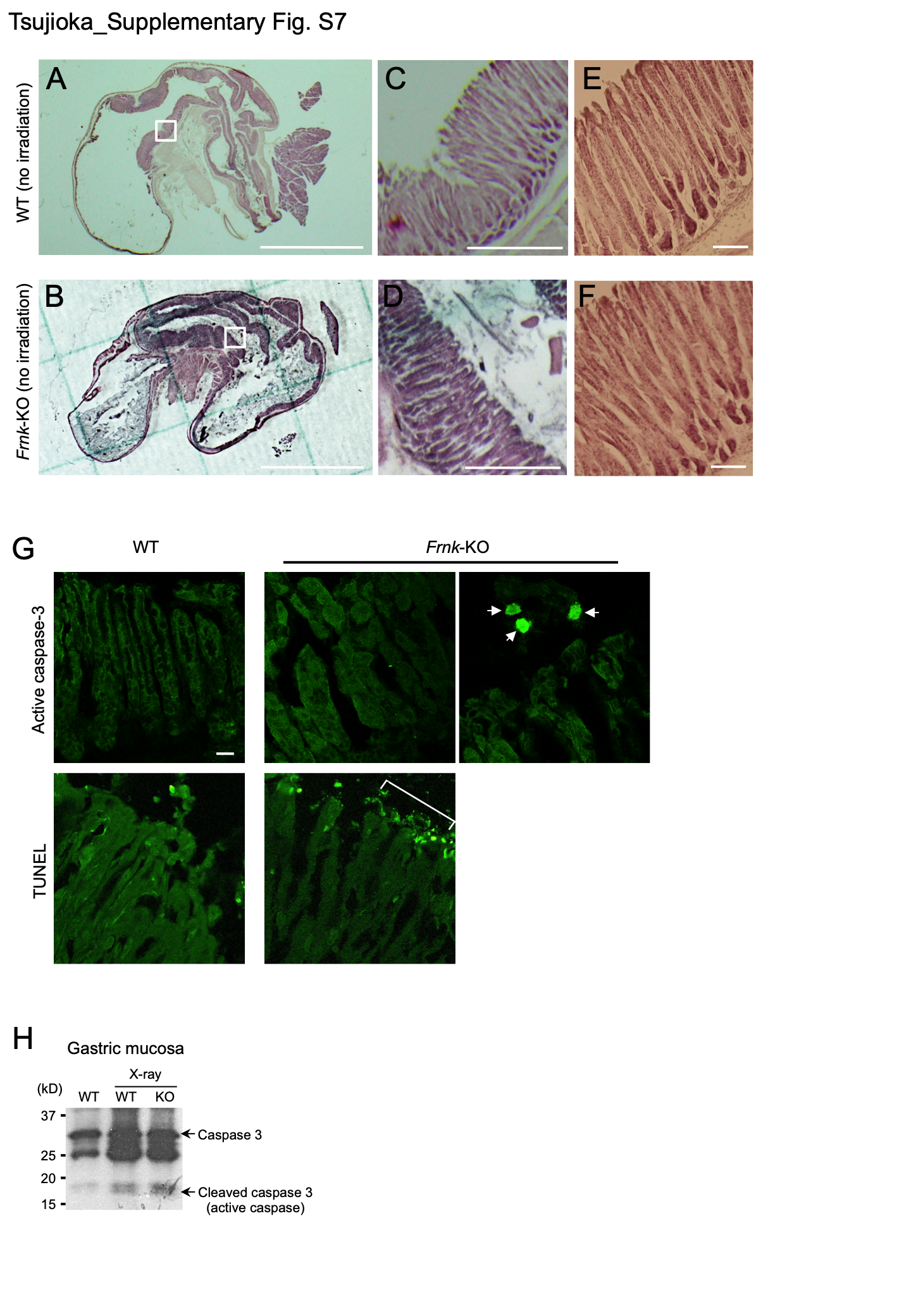


**Supplementary Fig. S7. Gastric analysis of WT and *Frnk*-KO mice.**

**A–F** HE staining of sliced sections of the nonirradiated stomachs of WT and *Frnk*-KO mice, which are the control specimens for Fig. 6D–I. (**C, D**) are magnified images of the boxed regions in (**A, B**), respectively. **G** Sliced sections of the gastric mucosa of irradiated (5 Gy) WT and *Frnk*-KO mice, fluorescently labelled with an antibody against active caspase 3 (upper panels) and subjected to TUNEL staining (lower panels). Arrows indicate stained cells that are not in the mucosal layer. TUNEL staining also showed strong fluorescence signals above the mucosal layer of *Frnk*-KO mouse stomachs (bracket). **H** Immunoblot analysis showing that active caspase 3 was similarly expressed in the gastric mucosa of irradiated WT and *Frnk*-KO mice. Scale bars, 5 mm (**A, B**), 0.5 mm (**C, D**), 0.1 mm (**E, F**), and 10 µm (**G**).

**
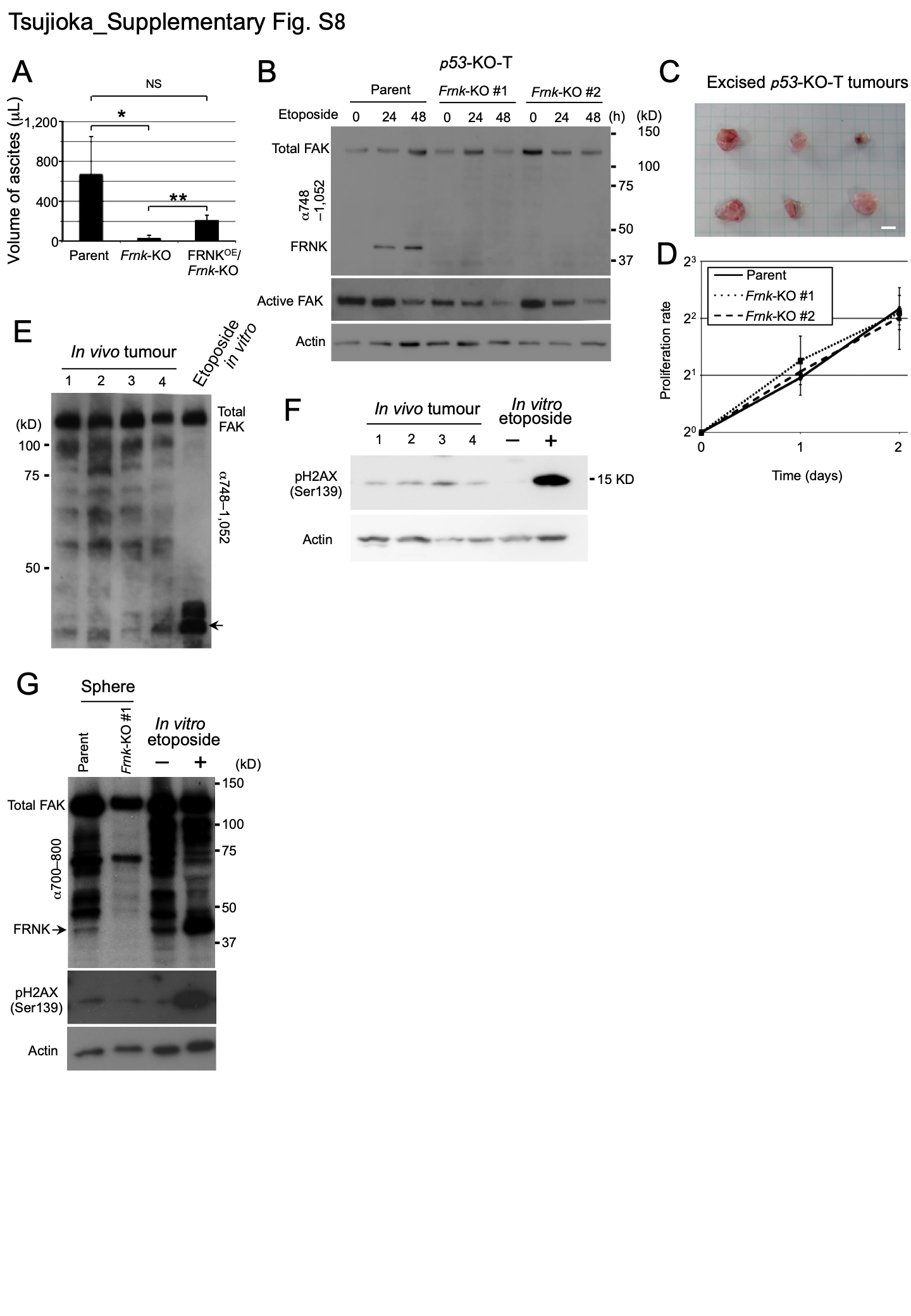
**

**Supplementary Fig. S8. Effects of FRNK in cancer progression in a mouse model.**

**A** Graph showing the volume of bloody ascites taken from the peritoneal cavities of mice in which parental, *Frnk*-KO, and FRNK^OE^/*Frnk*-KO CT26 cells were injected. The two-sided Student *t*-test was used for statistical analysis. *: *p* < 0.05, **: *p* < 0.01, NS: no significance. **B** Immunoblot analysis showing the expression of FRNK in *p53-*KO-T but not its *Frnk-*KO cell lines during etoposide (100 µM) treatment. All cell lines showed a reduction in the level of active FAK during etoposide treatment. **C** Macroscopic image of the excised *p53*-KO-T cell tumours at 16 days after intraperitoneal injection. Scale bar, 5 mm. **D** Graph showing equivalent proliferation rates among the parental and two *Frnk*-deficient *p53-*KO-T cell lines. Statistical analysis demonstrated no significant differences among the cell lines (n = 2). **E, F** *P53*-KO-T cells (3 × 10^5^ cells) were intraperitoneally injected into female syngeneic B6 mice, and tumours were excised after 16 days. Immunoblot analysis showing the expression levels of FRNK (arrow), total FAK (**E**), and pH2AX (**F**) *in vivo*, in four tumour masses. Cultured *p53*-KO-T cells treated with or without etoposide were used as positive and negative controls, respectively. **G** *P53*-KO-T cell derivatives (5 × 10^4^ cells) were 3D-cultured for 8 days to generate spheres. Immunoblot analysis showing the expression levels of FRNK (arrow) and pH2AX in *in vitro* tumour spheres of parental and *Frnk*-deficient *p53*-KO-T cells. Cultured *p53-*KO-T cells treated with or without etoposide were used as positive and negative controls, respectively.


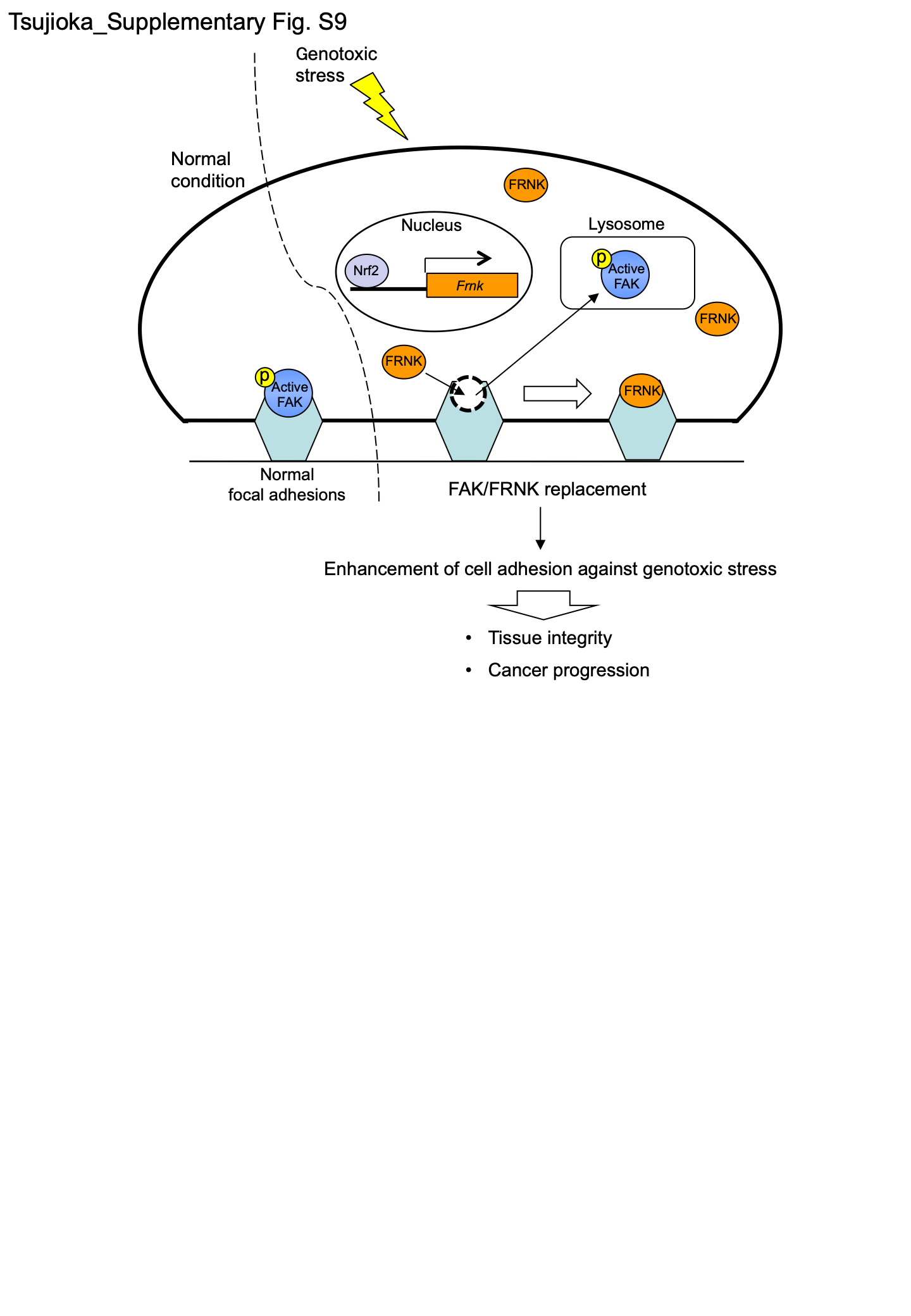


**Supplementary Fig. S9. Model showing the responses of focal adhesions to genotoxic stress.**

In normal conditions, active FAK acts as a component of focal adhesions. Upon genotoxic stress, however, Nrf2 drives the expression of the *Frnk* gene, whereas active FAK is reduced via lysosomal degradation. This results in FAK/FRNK replacement in focal adhesions, and this remodelling counteracts genotoxic stress in normal as well as pathological tissues by enhancing cell adhesion.


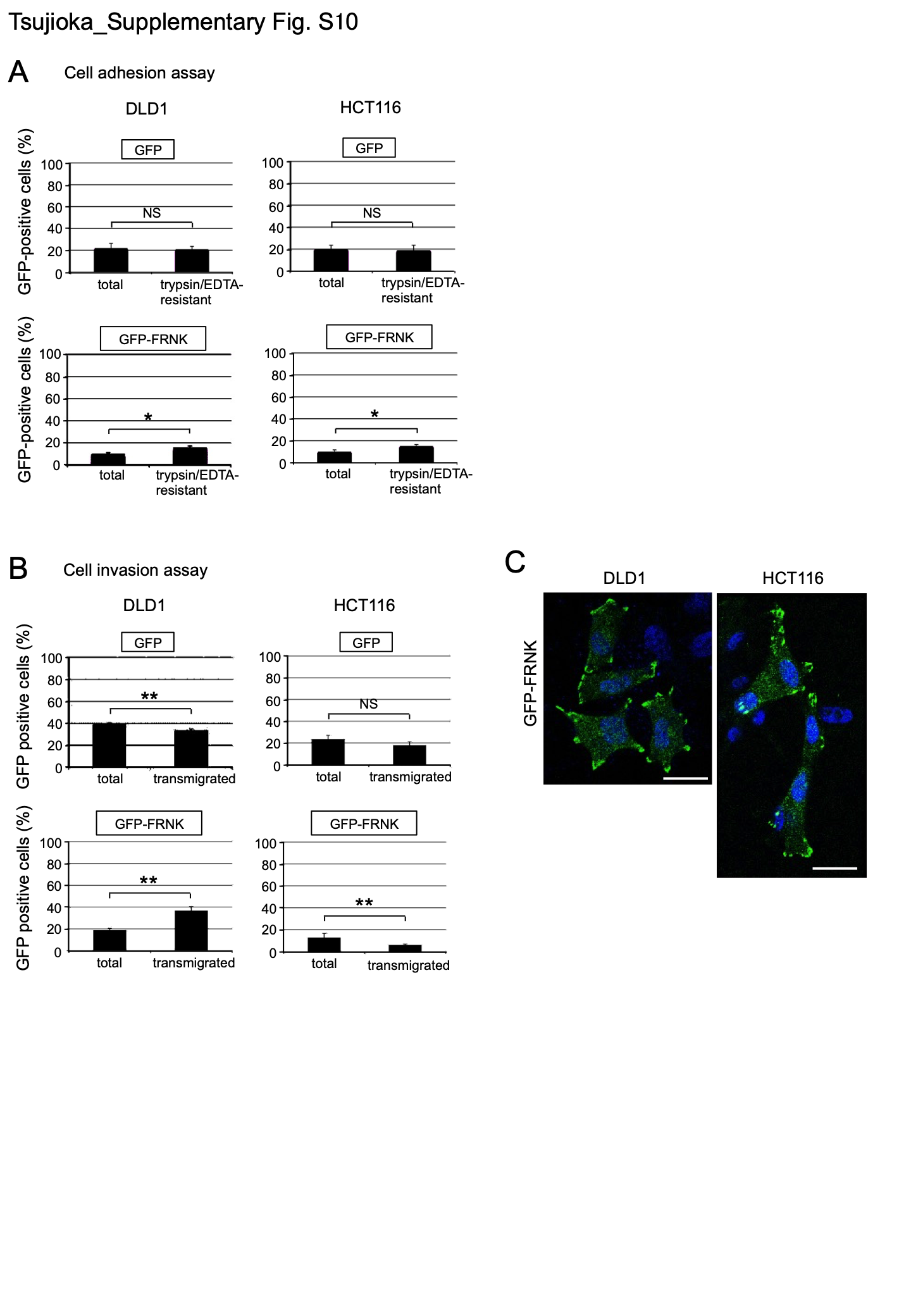


**Supplementary Fig. S10. Effects of FRNK in human colon cancer progression.**

**A, B** The indicated cell lines of human colon cancer were transfected with GFP and GFP-FRNK plasmids, resulting in a mixture of GFP-positive and GFP-negative cells. (**A**) Graphs showing ratios of GFP-positive populations in the mixtures (total) and in the cells remaining attached after trypsin-EDTA treatment. (**B**) Graphs indicating fractions of GFP-positive cells in the mixtures (total) and in the transmigrated cells. Note that reduction of the GFP-expressing transmigrated population in DLD1 cells was presumably due to cytotoxic effects of the transfection reagent. All data in the graphs are shown as means ± SD (n = 3). The two-sided Student *t*-test was used for statistical analysis. *: *p* < 0.05, **: *p* < 0.01, NS: no significance. **C** Confocal fluorescence images showing the subcellular localizations of GFP-FRNK in the indicated cell lines. Scale bars, 20 µm


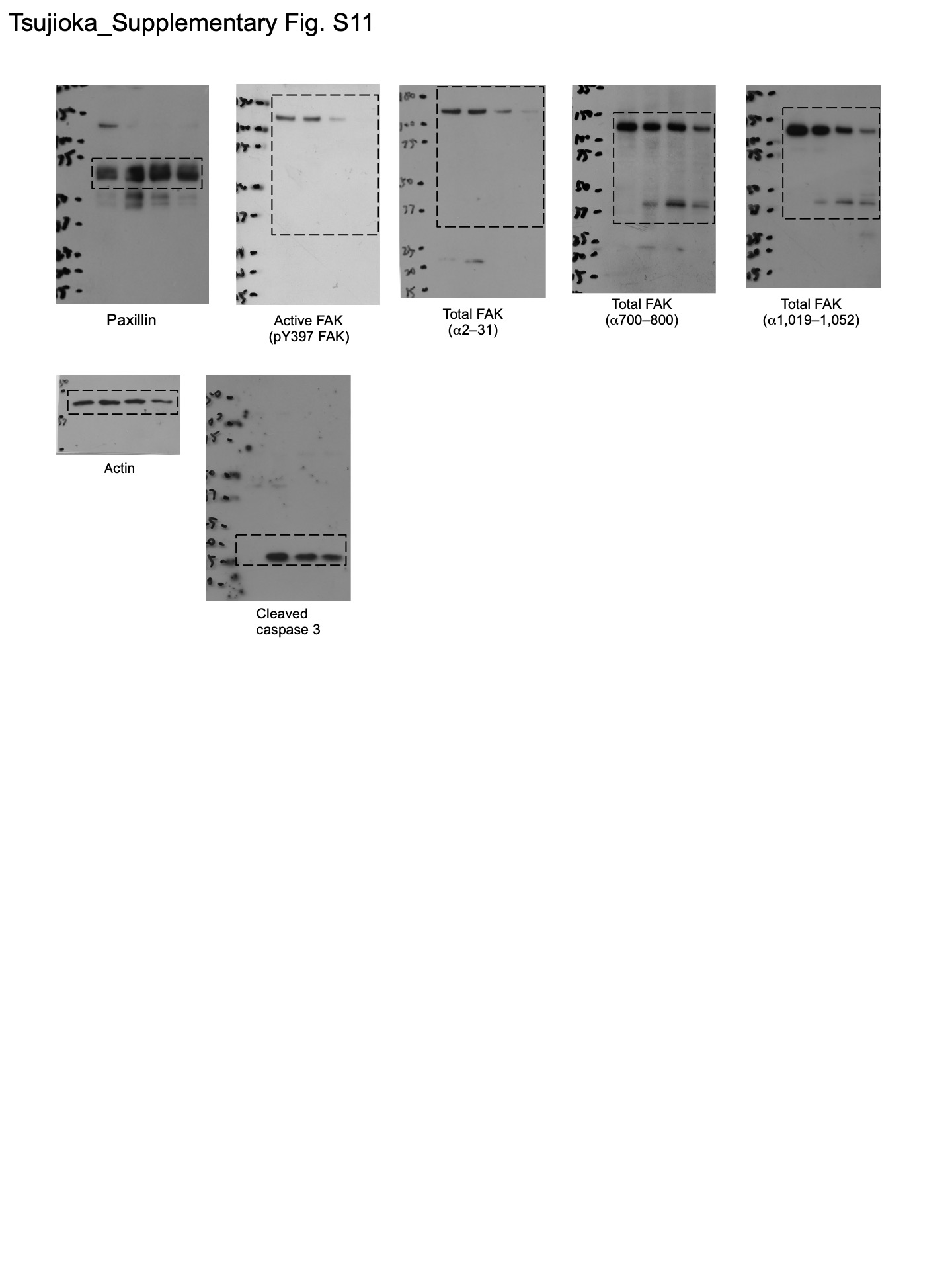


**Supplementary Fig. S11. Original immunoblots for Fig. 1F.**

**
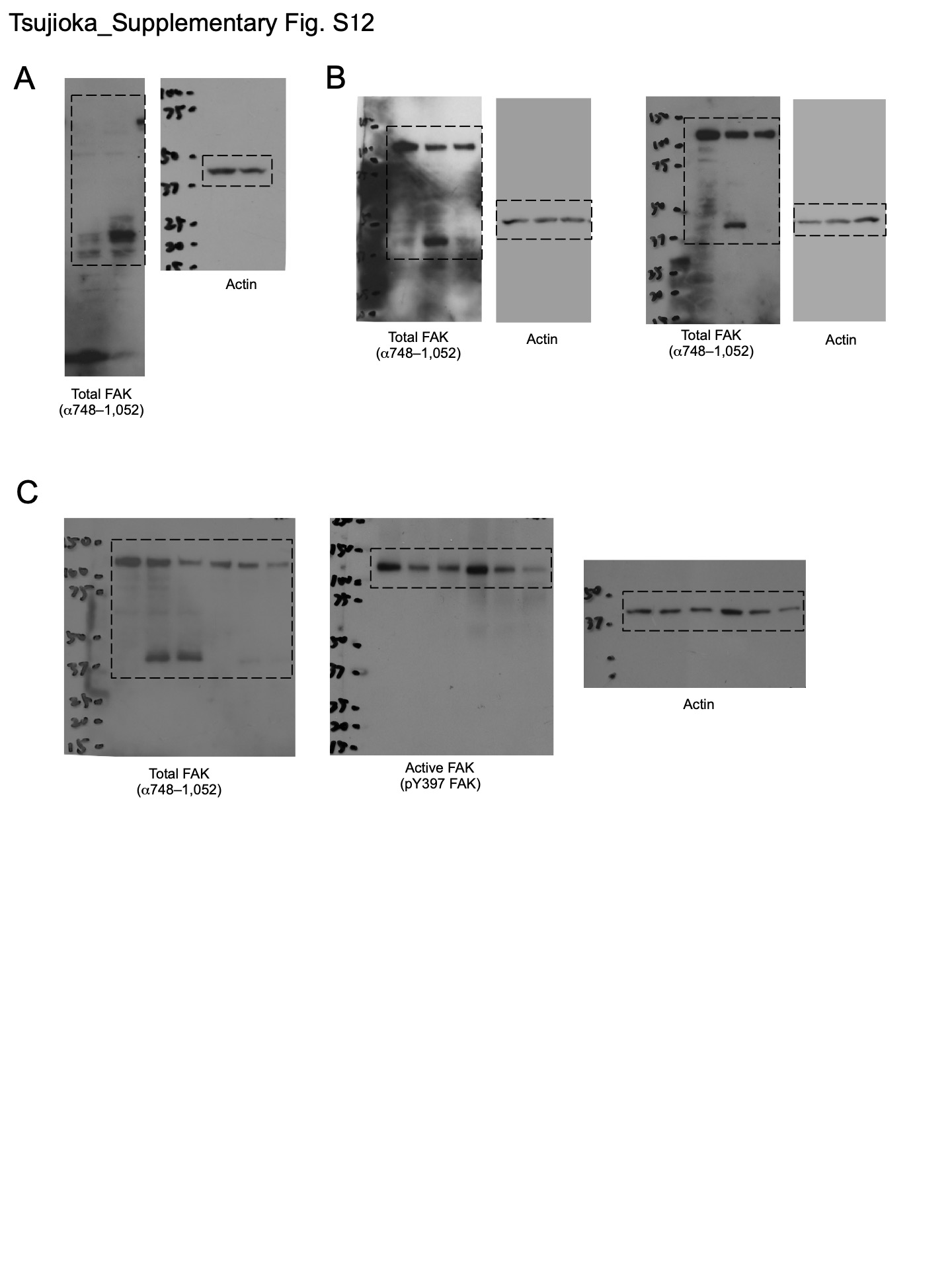
**

**Supplementary Fig. S12. Original immunoblots for Fig. 2.**

**A** is for Fig. 2A. **B** is for Fig. 2D. **C** is for Fig. 2G.


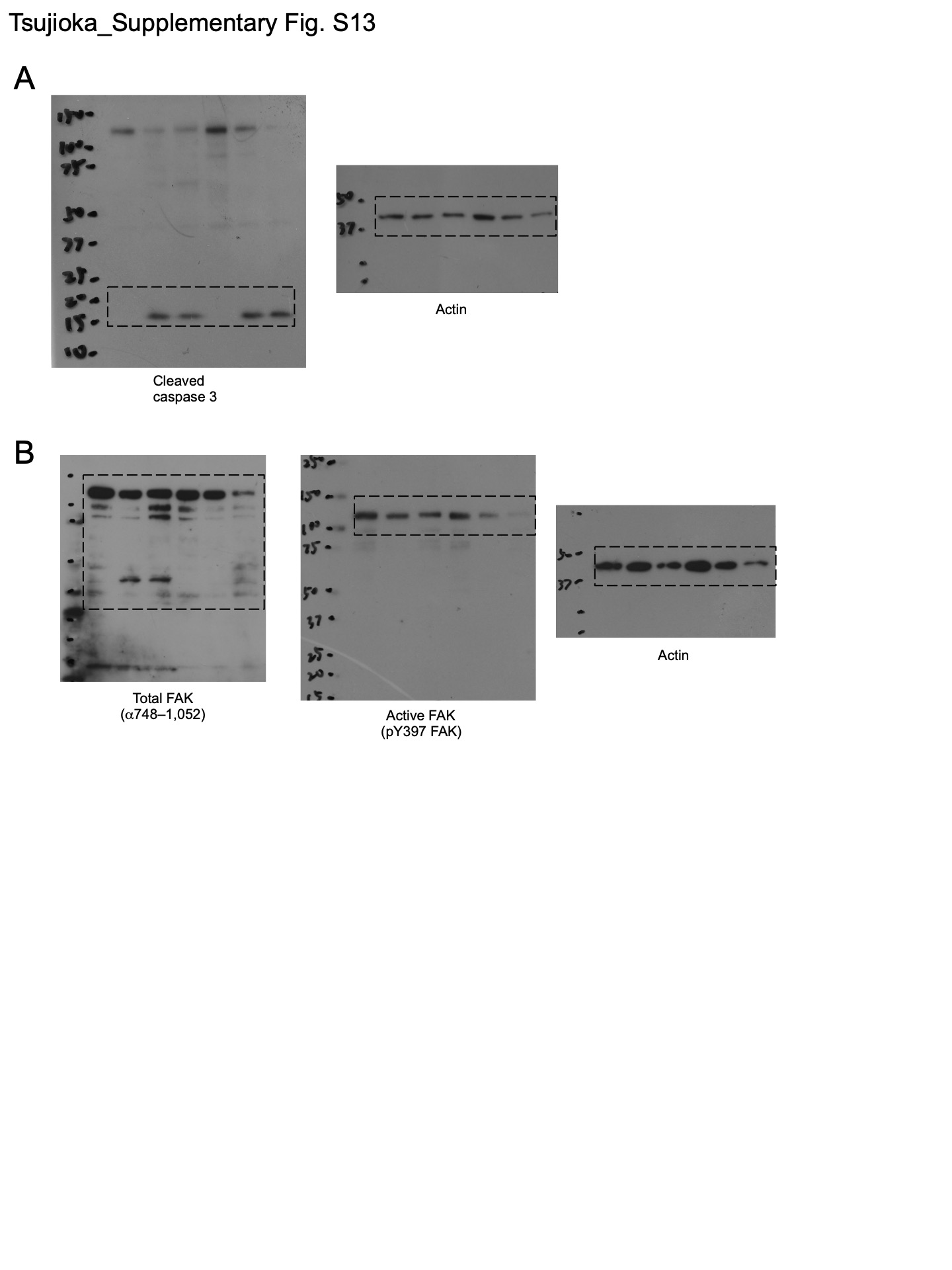


**Supplementary Fig. S13. Original immunoblots for Fig. 3.**

**A** is for Fig. 3D. **B** is for Fig. 3E.


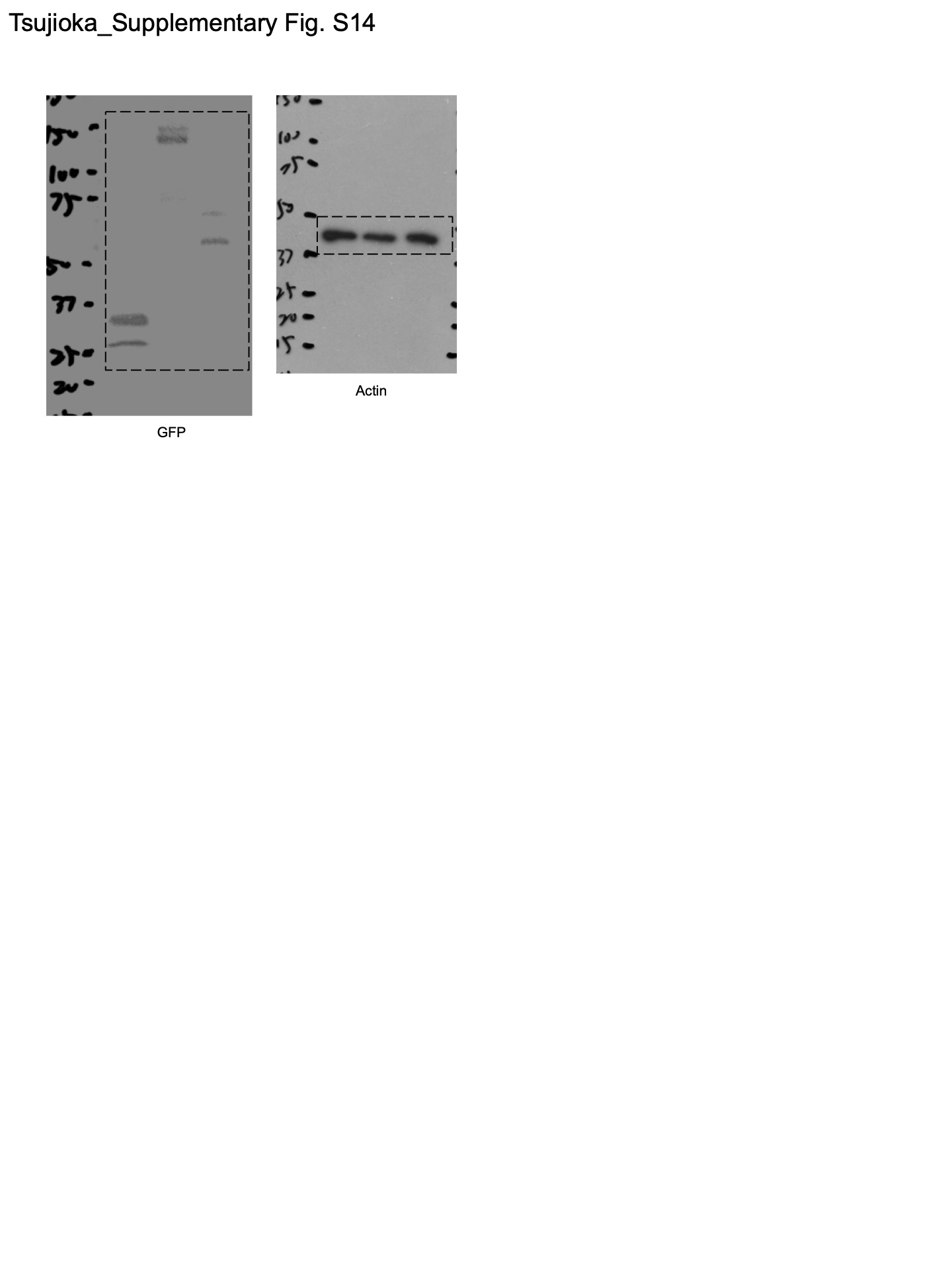


**Supplementary Fig. S14. Original immunoblots for Fig. 4A.**

**
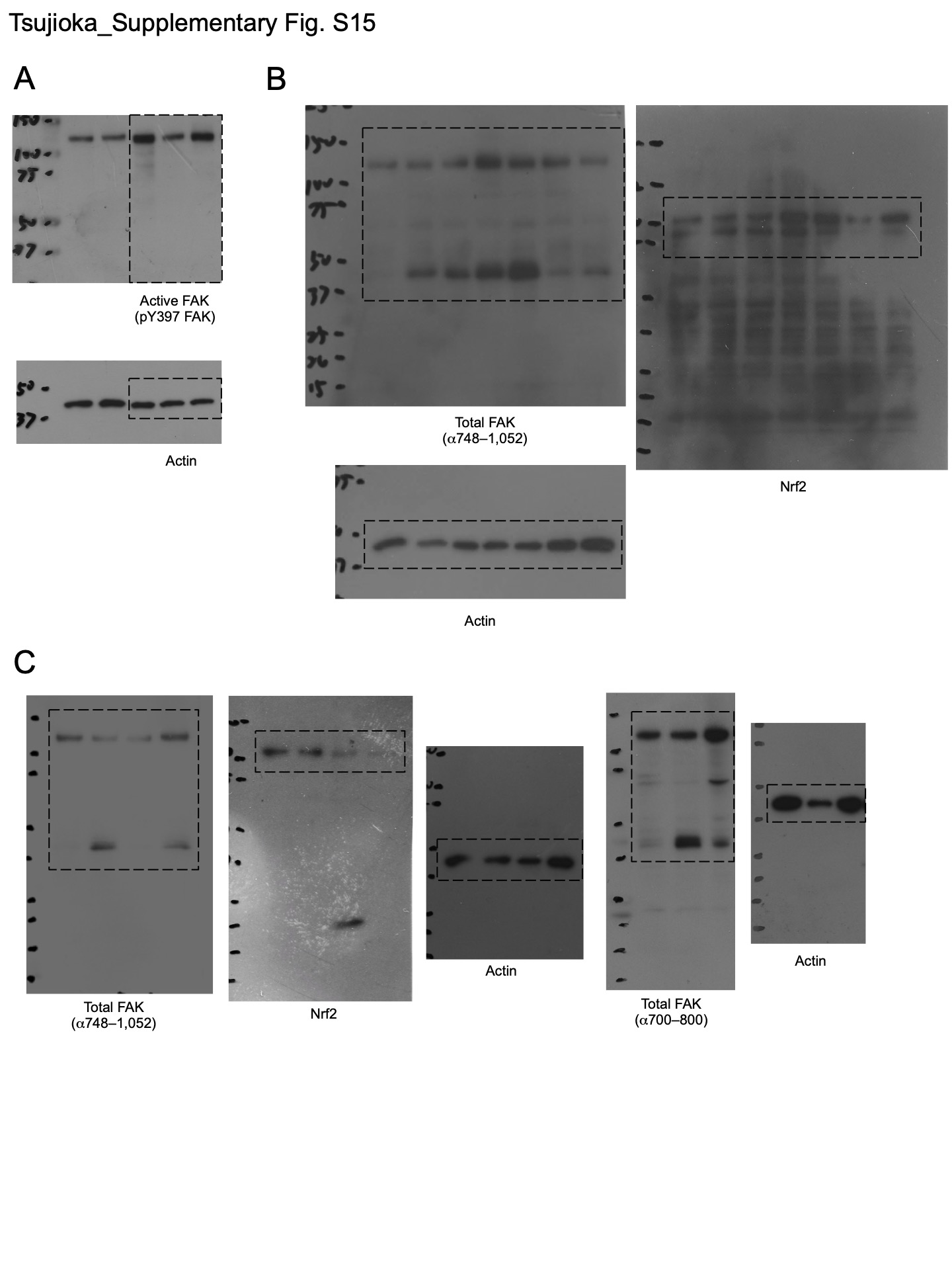
**

**Supplementary Fig. S15. Original immunoblots for Fig. 5.**

**A** is for Fig. 5A. **B** is for Fig. 5E. **C** is for Fig. 5F.


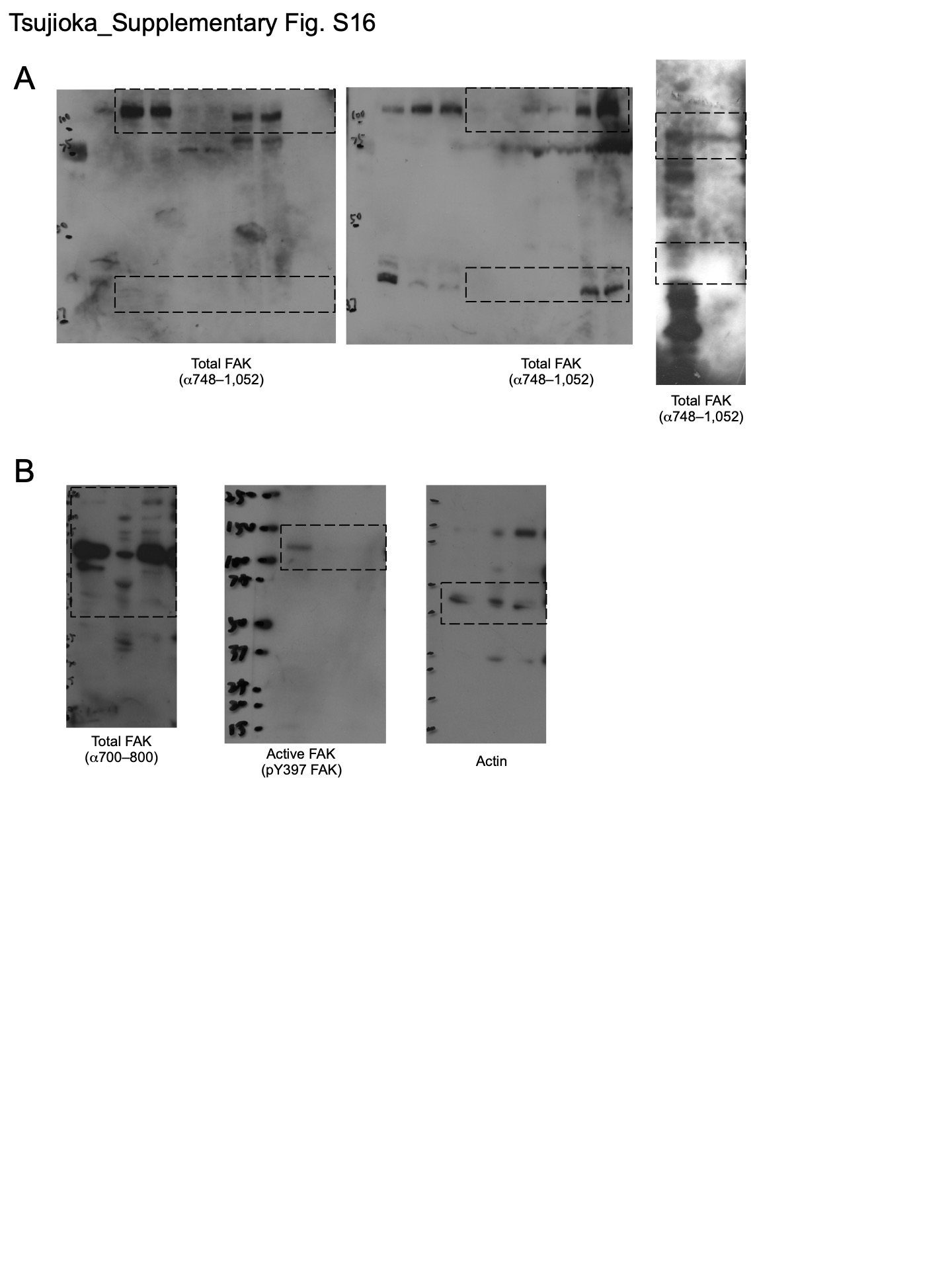


**Supplementary Fig. S16. Original immunoblots for Fig. 6.**

**A** is for Fig. 6A. **B** is for Fig. 6B.


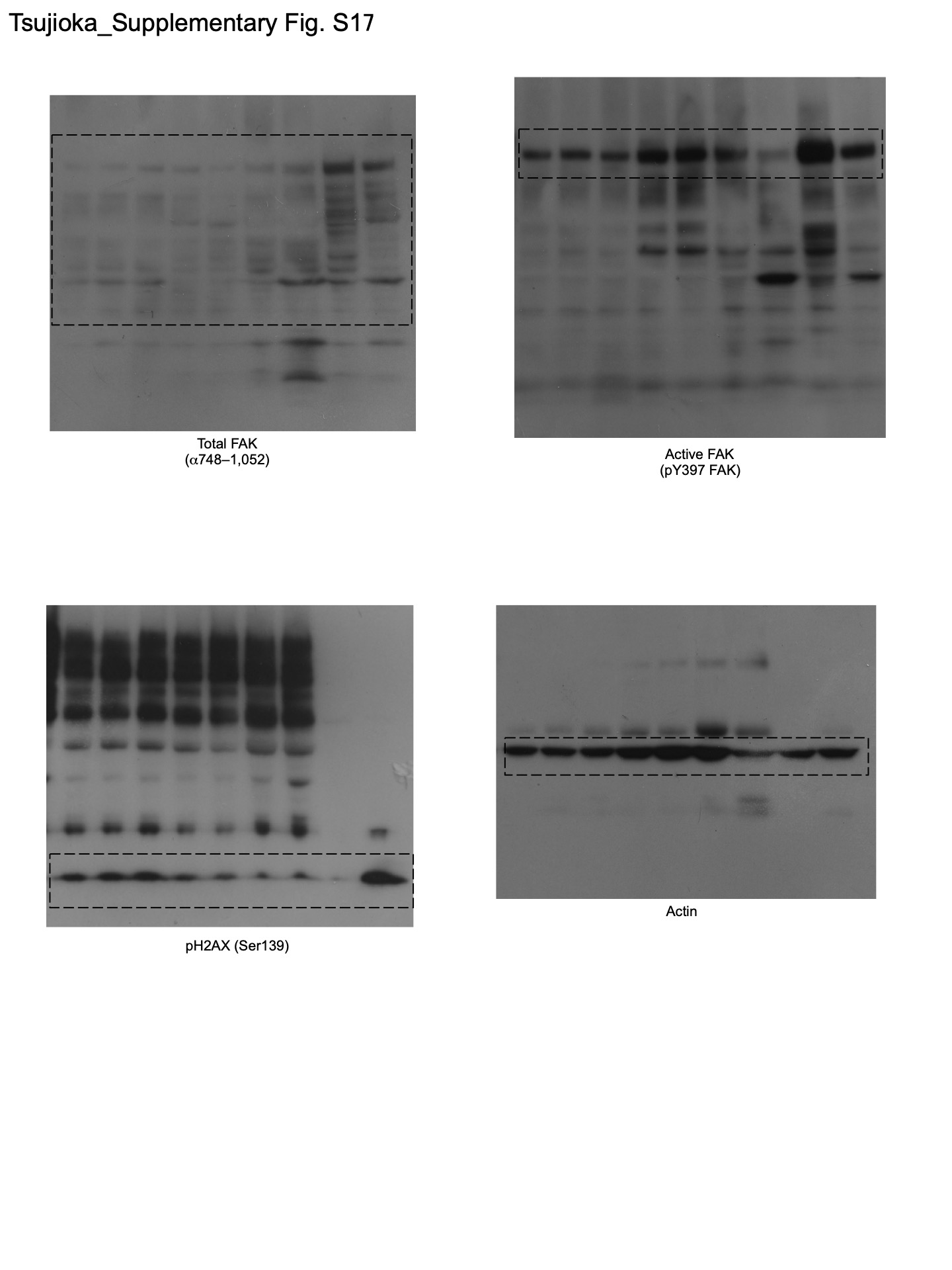


**Supplementary Fig. S17. Original immunoblots for Fig. 7A.**

**
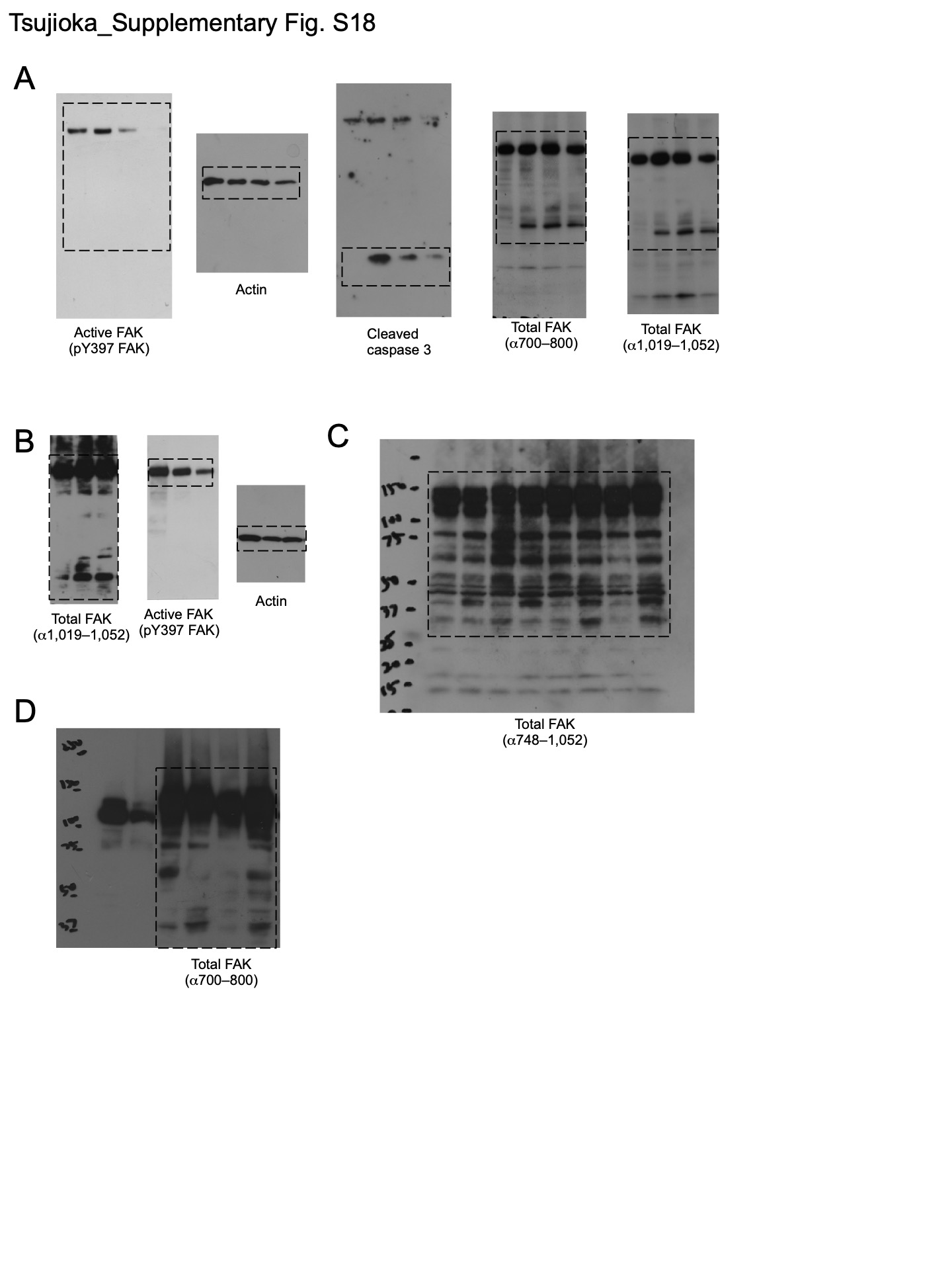
**

**Supplementary Fig. S18. Original immunoblots for Supplementary Fig. S2.**

**A** is for Supplementary Fig. S2A. **B** is for Supplementary Fig. S2B. **C** is for Supplementary Fig. S2D. **D** is for Supplementary Fig. S2E.


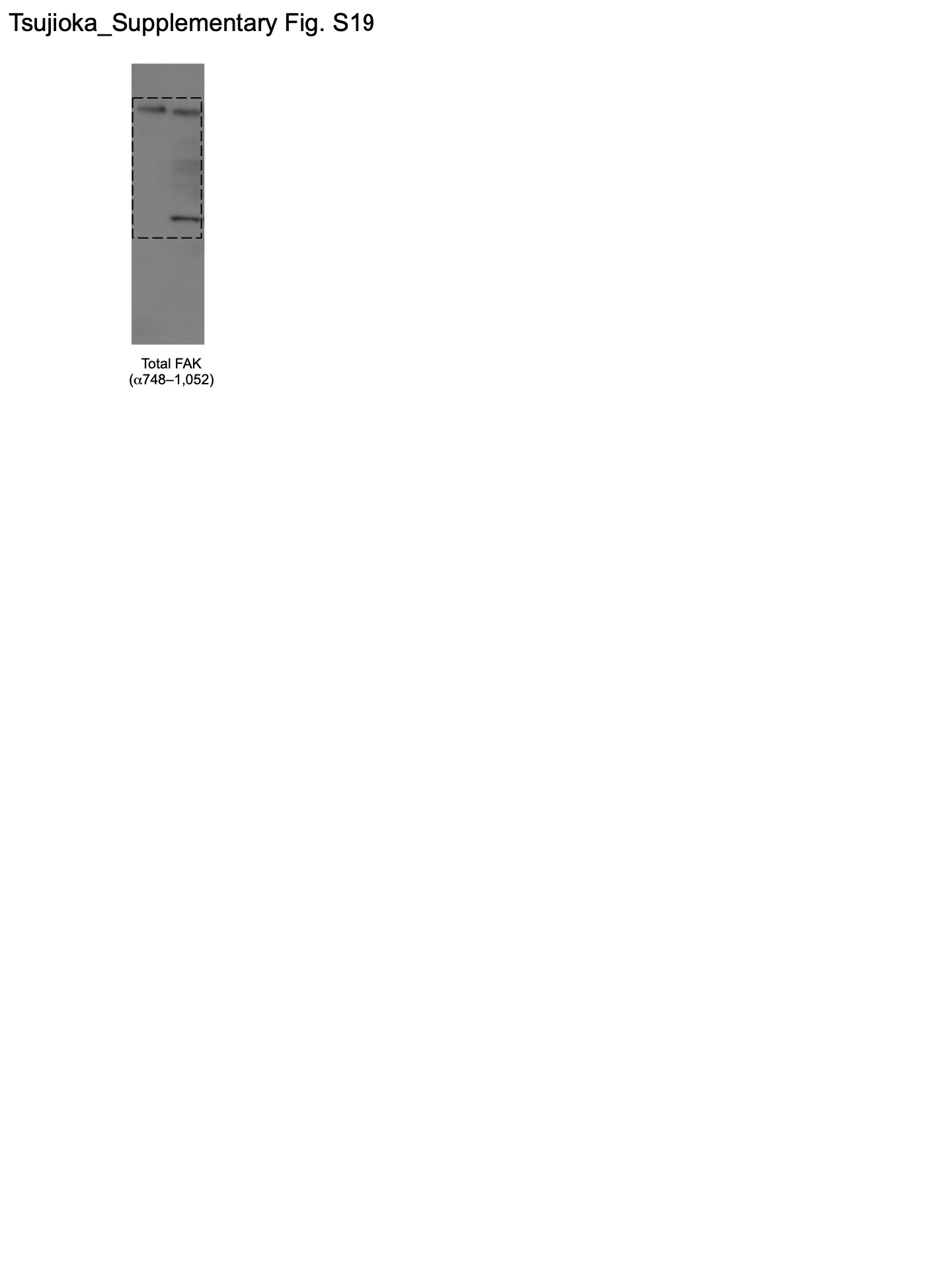


**Supplementary Fig. S19. Original immunoblots for Supplementary Fig. S3F.**

**
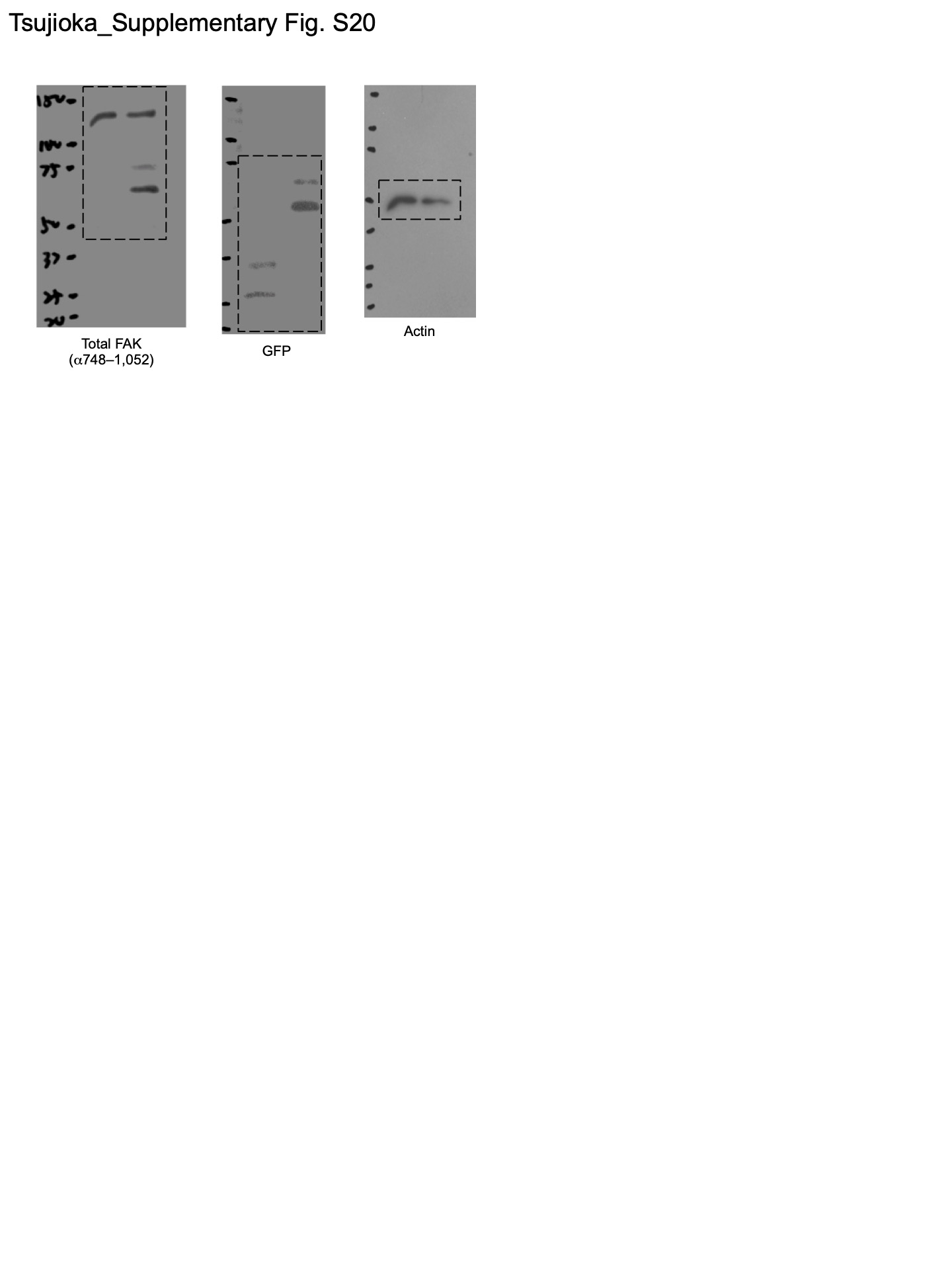
**

**Supplementary Fig. S20. Original immunoblots for Supplementary Fig. S5B.**

**
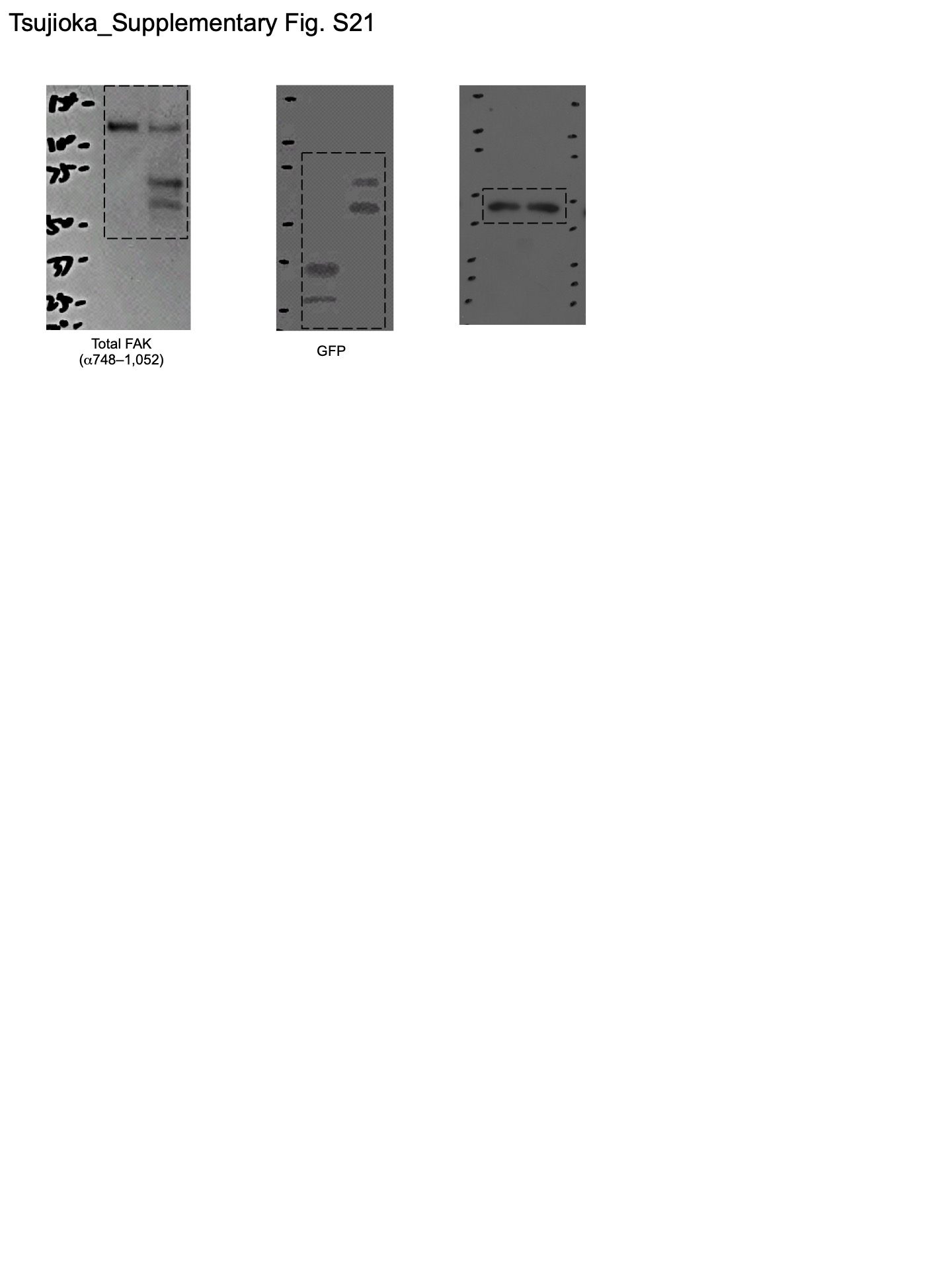
**

**Supplementary Fig. S21. Original immunoblots for Supplementary Fig. S6B.**

**
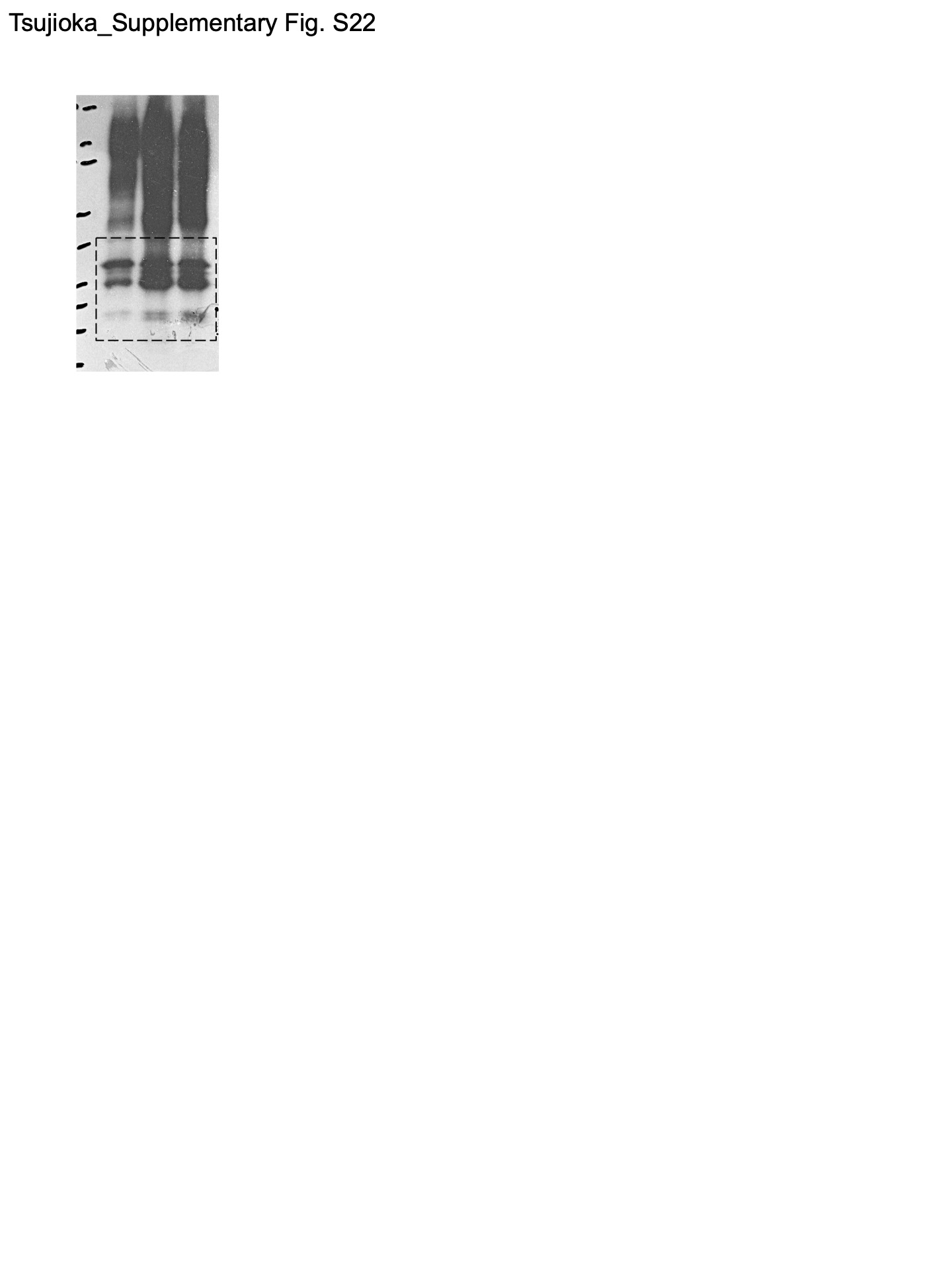
**

**Supplementary Fig. S22. Original immunoblots for Supplementary Fig. S7H.**

**
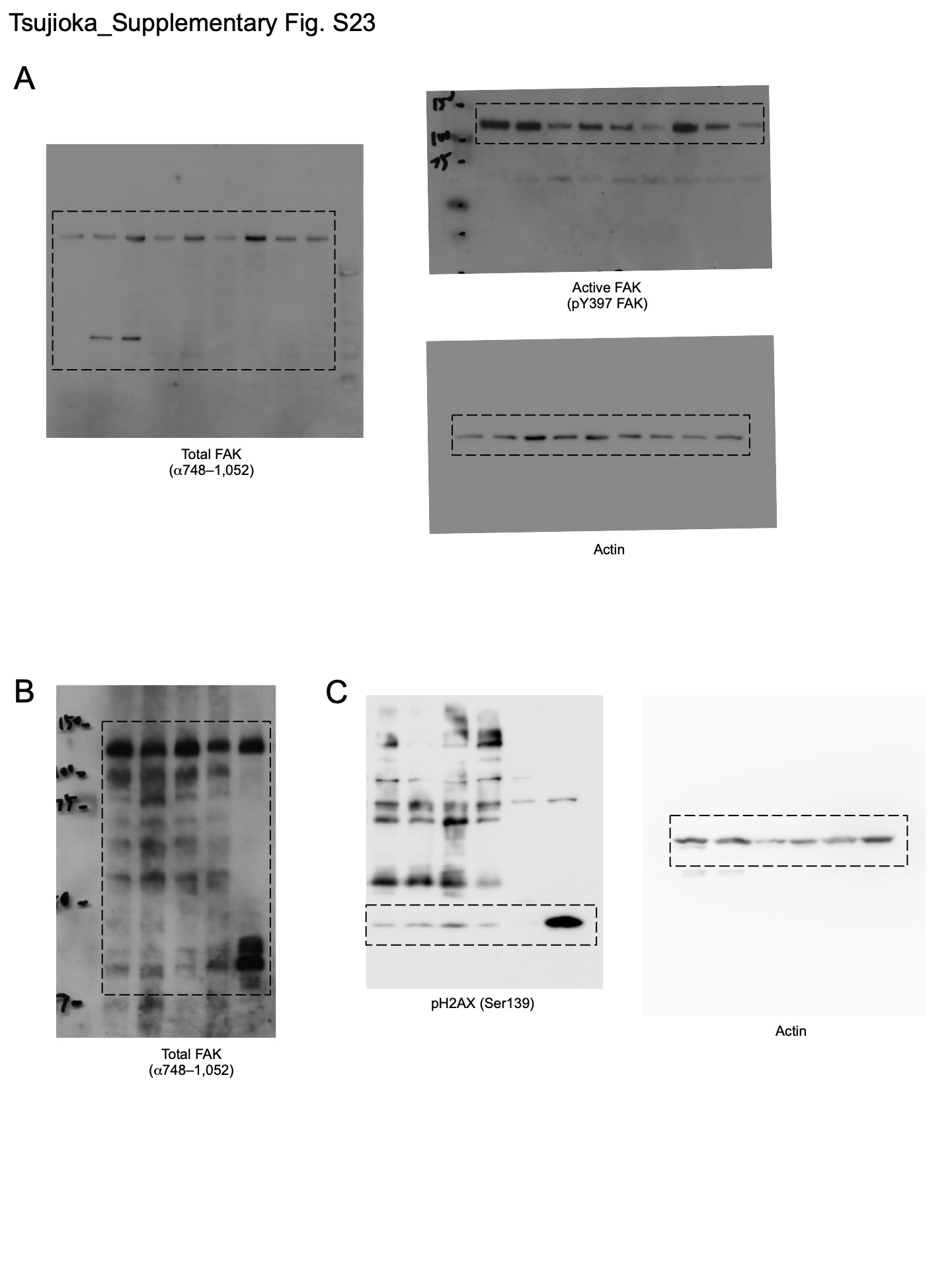
**

**
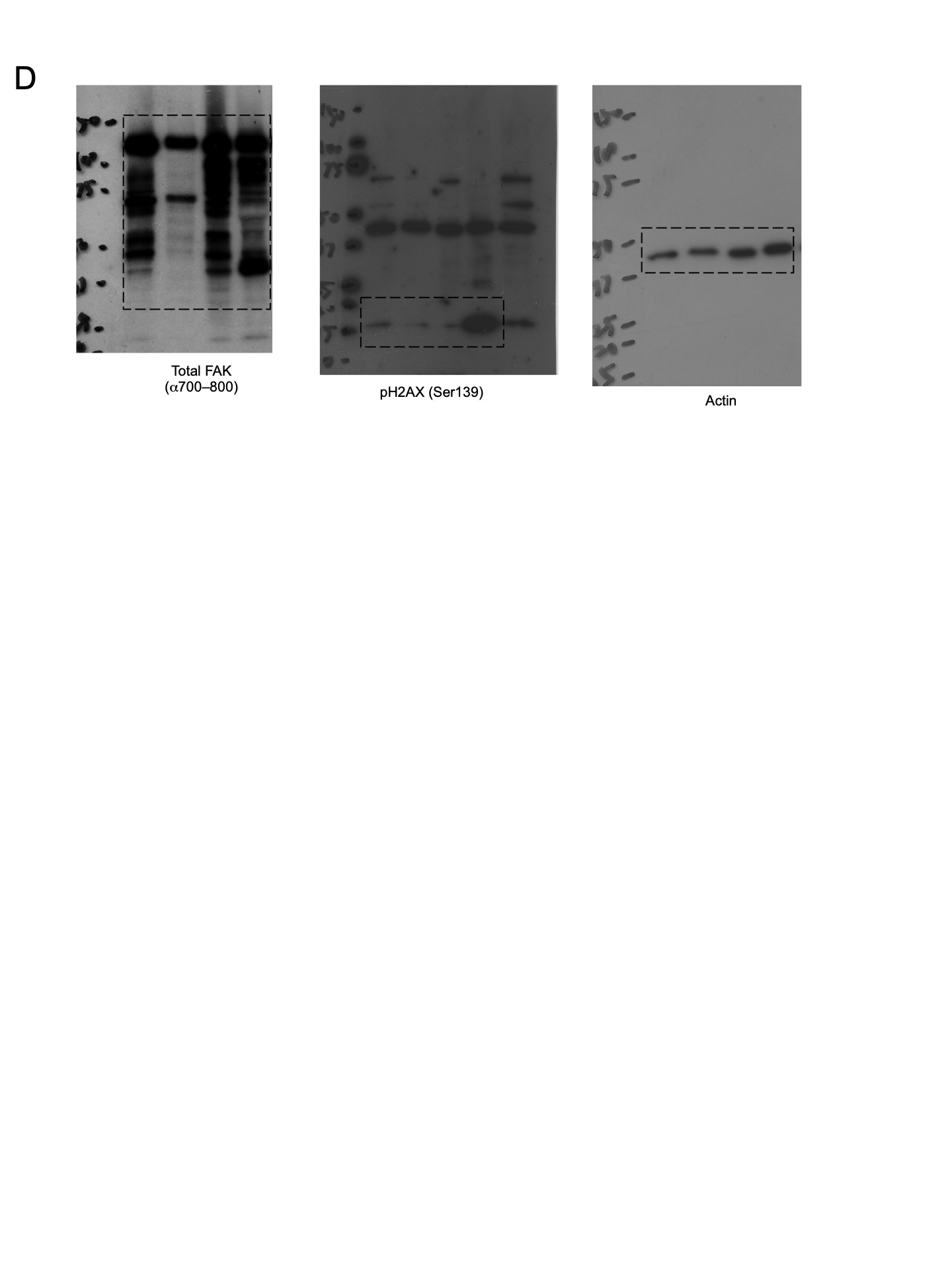
**

**Supplementary Fig. S23. Original immunoblots for Supplementary Fig. S8.**

**A** is for Supplementary Fig. S8B. **B** is for Supplementary Fig. S8E. **C** is for Supplementary Fig. S8F. **D** is for Supplementary Fig. S8G.

**Supplementary Table S1. List of antibodies used in this study.**

**Supplementary Table S2. List of reagents used in this study.**

**Supplementary Table S3. List of cell lines in which FRNK expression was analysed.**

“+” indicates the detection of FRNK after etoposide treatment.

**Supplementary Table S4.** Table showing information regarding the biopsy samples.

“+” indicates the detection of the *FRNK* gene expression.
